# Efficacy and safety of Tongxinluo in the treatment of stroke: a systematic review and meta-analysis of randomized controlled trials

**DOI:** 10.3389/fphar.2025.1573069

**Published:** 2025-07-21

**Authors:** Zhixin Wu, Jiamei Fu, Yumeng Zhou, Yabin Zhou

**Affiliations:** ^1^ Heilongjiang University of Chinese Medicine, Harbin, Heilongjiang, China; ^2^ The First Affiliated Hospital of Heilongjiang University of Chinese Medicine, Harbin, Heilongjiang, China

**Keywords:** tongxinluo, adjunctive treatment, traditional Chinese medicine, stroke, meta-analysis

## Abstract

**Objective:**

To evaluates the efficacy and safety of Tongxinluo in treating stroke.

**Methods:**

PubMed, Web of Science, Cochrane Library, Embase, China National Knowledge Infrastructure and Wanfang databases were performed to search literature from 2000 to 2024. Randomized controlled trials evaluating Tongxinluo for stroke were included. The primary outcomes were efficacy and safety. Sensitivity and subgroup analyses were conducted to assess result stability and identify sources of heterogeneity. All analyses were conducted using Review Manager 5.4 and STATA 15.1.

**Results:**

Fifty-one RCTs including 9,577 participants for analysis. Tongxinluo significantly outperformed the control group in efficacy [RR = 1.20, 95% CI (1.16, 1.25)]. Adverse event incidence between groups showed no significant difference [RR = 1.01, 95% CI (0.90, 1.12)]. Additionally, Tongxinluo significantly improved NIHSS, total cholesterol (TC), and serum hypersensitive C-reactive protein (hs-CRP) levels in stroke individuals.

**Conclusion:**

Tongxinluo, as an adjunctive treatment for stroke, offers superior clinical efficacy compared to conventional treatments without increasing adverse event risk. However, due to study limitations, further multicenter, large-sample RCTs are required to confirm.

**Systematic Review Registration:**

https://www.crd.york.ac.uk/PROSPERO/.

## 1 Introduction

Stroke is an acute neurological disorder caused by cerebrovascular disease, leading to impaired cerebral blood circulation. It can be ischemic (cerebral infarction) or hemorrhagic (including intracerebral hemorrhage, subarachnoid hemorrhage, and others). Stroke has a high incidence, recurrence, disability, mortality, and economic burden. It is the second leading cause of death worldwide and the primary cause of adult disability. In developing countries, stroke-related deaths account for about two-thirds of global deaths (Tu et al., 2023a). The 2013–2020 Stroke High-Risk Population Screening and Intervention Program in China found that the average age of first stroke onset for individuals aged 40 and older ranged from 60.9 to 63.4 years, with over 66.6% of patients aged 40–64 years (Tu et al., 2023b). Although enrollment data standardization methods may vary, the findings suggest a younger age of stroke onset in China. Stroke incidence is higher in men than women, with the male incidence rate increasing annually from 2013 to 2020, while the female rate remained stable (Guzik and Bushnell, 2017). Primary stroke treatments, including thrombolysis, surgery, and pharmacological therapy, are associated with adverse effects. Efforts to improve patients’ post-treatment quality of life are needed. Traditional Chinese medicine (TCM), with its long history in stroke treatment, is referred to as “Zhongfeng.” This term, first mentioned in the *Jinkui Yaolue* (Essential Prescriptions of the Golden Cabinet), describes symptoms such as hemiplegia, numbness, facial drooping, speech difficulties, and, in severe cases, collapse and loss of consciousness. The *Jinkui Yaolue* also recorded the use of Chinese herbal decoctions for stroke treatment (Zhong et al., 2020).

Tongxinluo is an antioxidant that protects the blood-brain barrier, promotes axonal plasticity, stabilizes vulnerable plaques, inhibits delayed neuronal death, and improves stroke prognosis. Approved by the China Food and Drug Administration in 1996, it is widely used in China to treat acute unstable angina (Wu et al., 2006). Tongxinluo contains 12 traditional Chinese herbs: *Boswellia sacra Flück.,Dalbergia odorifera T.C.Chen, Panax ginseng C.A.Mey.,Paeonia lactiflora Pall.,Ziziphus jujuba Mill.,Santalum album L.,Blumea balsamifera (L.) DC.,Cucumis melo L.,Salvia miltiorrhiza Bunge, Girardinia diversifolia subsp. diversifolia, Eupolyphaga sinensis Walker, Scolopendra subspinipes mutilans* L. Koch (Chen et al., 2024). Pharmacological studies show that Tongxinluo has vasodilatory, antiplatelet, anticoagulant, thrombolytic, and lipid-lowering effects, protecting against cerebral ischemic injury (Bu et al., 2008).

A 2013 study by Jianjun Wen demonstrated that Tongxinluo effectively treats acute cerebral infarction. Involving 278 patients, it showed significant improvement in NIHSS scores after 4 weeks of treatment (Wen et al., 2013). Similarly, a 2008 study by Junke Cui, involving 112 patients, found significant improvements in acute cerebral infarction outcomes after 30 days of treatment (Cui et al., 2008).

However, Zhou et al. (2016) reported no significant improvement in NIHSS scores among stroke patients treated with Lan et al. (2013) likewise found no substantial benefit in overall therapeutic outcomes. These findings indicate that existing evidence remains inadequate to substantiate the clinical value of Tongxinluo in stroke management, with limited support from evidence-based studies. To address this gap, we conducted the first systematic review and meta-analysis to assess its efficacy, safety, and potential moderating factors in the treatment of stroke.

## 2 Methods and materials

### 2.1 Protocol and registration

Following the PRISMA guidelines and registering the study in PROSPERO (registration number CRD42024621065).

### 2.2 Search method

Systematic searches were conducted up to October 2024 in PubMed (((“tongxinluo” [Supplementary Concept]) OR (Tongxinluo)) AND ((“Stroke, Lacunar” [Mesh]) OR (((((((((((((((((Strokes) OR (Cerebrovascular Accident)) OR (Cerebrovascular Accidents)) OR (Cerebral Stroke)) OR (Cerebral Strokes)) OR (Cerebrovascular Apoplexy)) OR (Brain Vascular Accident)) OR (Brain Vascular Accidents)) OR (Cerebrovascular Stroke)) OR (Cerebrovascular Stroke)) OR (Apoplexy)) OR (CVA)) OR (CVAs)) OR (Acute Stroke)) OR (Acute Strokes)) OR (Acute Cerebrovascular Accident)) OR (Acute Cerebrovascular Accidents)))) AND (random*), Embase, Cochrane, Web of Science, VIP Database, Chinese Biomedical Literature Database, China National Knowledge Infrastructure (CNKI), and Wanfang databases to explore the use of Tongxinluo in stroke treatment. Search terms included “Tongxinluo,” “Stroke,” “Cerebrovascular Accident,” “Brain Vascular Accident,” and “randomized controlled trials,” with no linguistic or geographical restrictions. Additionally, we manually reviewed references from identified articles meeting the inclusion criteria to supplement the search. The search details were depicted in Supplementary Table S1.

### 2.3 Study selection

Eligible RCTs were included following a screening and evaluation of the literature through the title, abstract, and full text.

Inclusion criteria:

(a) Participants: Adults (≥18 years) with clinically diagnosed stroke, with no gender or racial restrictions.
(b) Intervention and comparison: Conventional with Tongxinluo capsules and conventional treatments.
(c) More than one outcome **exists**, including primary outcomes such as the clinical response rate and adverse event rate. Secondary outcomes-NIHSS, total cholesterol (TC), triglyceride (TG), HDL, LDL, TNF-α, hs-CRP, IL-6, IL-18, nitric oxide (NO), Barthel Index, whole blood viscosity (high and low cut), plasma viscosity, erythrocyte ratio, plasma fibrinogen, quality of life score, Fugl-Meyer (FM), MMSE, inflammatory myofibroblastic tumor (IMT), plaque area, platelet aggregation function.

The main metabolites of Tongxinluo are shown in Supplementary Table S2.

Exclusion criteria:

(a) Non-RCTs, retrospective studies, animal studies, and review literature.
(b) Patients with other cardiovascular conditions. For example, atrial fibrillation, coronary heart disease, heart failure, cardiomyopathy, valvular heart disease, and hypertension.
(c) Studies where the intervention group received alternative traditional Chinese medicine treatments, including Chinese patent medicines, pills, injections, acupuncture, massage, or auricular acupuncture.
(d) Studies with inaccurate data, incomplete outcome measurements, or lack of access to original data.
(e) Duplicate publications.

### 2.4 Composition and preparation methods of tongxinluo

Tongxinluo contains 12 traditional Chinese medicinal ingredients: *Boswellia sacra Flück.*, *Dalbergia odorifera T.C*. *Chen*, *Panax ginseng C.A. Mey.*, *Paeonia lactiflora Pall.*, *Ziziphus jujuba Mill.*, *Santalum album L.*, *Blumea balsamifera (L.) DC.*, *Cucumis melo L.*, *Salvia miltiorrhiza Bunge*, *Girardinia diversifolia subsp. diversifolia*, *E. sinensis Walker*, and *S. subspinipes mutilans L. Koch*.

The extraction of each herbal component in the Tongxinluo capsules used in this study strictly followed national pharmacopeial standards and modern Chinese medicine preparation protocols. Methods were determined based on the characteristics of each herb and the nature of its active constituents, as outlined below:

Frankincense (*Boswellia sacra Flück.*), dalbergia odorifera (*Dalbergia odorifera T.C.Chen*), and sandalwood (*Santalum album L.*): These herbs were processed using ethanol reflux extraction combined with steam distillation. After pulverization, the materials were extracted twice with six times the volume of 85% ethanol for 1.5 h each. The combined ethanol extracts were recovered and concentrated to obtain a resinous extract rich in lipophilic constituents. The residues were then subjected to steam distillation to collect volatile oils, mainly containing monoterpenes and sesquiterpenes.

Ginseng (*Panax ginseng C.A.Mey.*): Extracted by water decoction. Ginseng slices were soaked in ten times the volume of water for 0.5 h and decocted three times (2 h, 1.5 h, 1.5 h). The combined decoctions were filtered and concentrated into a clear extract with a relative density of 1.10–1.15 at 60°C, or further processed by spray drying to obtain ginseng extract powder containing ginsenosides and polysaccharides.

White peony root (*Paeonia lactiflora Pall.*) and sour jujube seed (*Ziziphus jujuba Mill.*): Extracted by water decoction. The herbs were decocted three times with eight times the volume of water (1.5 h each time). The combined extracts were filtered and concentrated. Ethanol precipitation was used to remove impurities (adjusted to 60%–70% ethanol), followed by standing and collection of the supernatant. Ethanol was recovered, and the remaining solution was dried to yield purified extracts rich in paeoniflorin, saponins, and flavonoids.

Borneo (*Blumea balsamifera (L.) DC*.) (natural borneol): As a sublimable crystalline substance, it was directly ground to a fine powder (passed through a No. 9 sieve, 200 mesh) without extraction.

Medicinal leech (*Cucumis melo L.*), cicada molt (*Salvia miltiorrhiza Bunge*), Chinese scorpion (*Girardinia diversifolia subsp. diversifolia*), Chinese cockroach (*E. sinensis* Walker), and centipede (*S. subspinipes mutilans* L. Koch): These animal-derived ingredients are rich in proteins, enzymes, and bioactive peptides and are heat-sensitive. A low-temperature ultrafine grinding technique was used. After selection and cleaning, the materials were dried below 60°C, sterilized using either cobalt-60 irradiation or steam flash sterilization, and ground into ultrafine powder (≤45 μm, passed through a 325-mesh sieve) to preserve biological activity.

The processed products were mixed according to the prescription ratio: refined water-ethanol extracts (or dried powder) of ginseng, white peony root, and sour jujube seed; ethanol extracts and volatile oils (often cyclodextrin-encapsulated) of frankincense, dalbergia odorifera, and sandalwood; fine powder of borneo; and ultrafine powders of centipede, medicinal leech, cicada molt, Chinese scorpion, and Chinese cockroach. Appropriate excipients were added, and the blend was processed through granulation, drying, sizing, and capsule filling. All procedures complied with Good Manufacturing Practice (GMP) standards.

### 2.5 Data extraction

Two investigators collected data independently, and the information including:

(a) Publication details;
(b) Study characteristics;
(c) Participant characteristics;
(d) Intervention details;
(e) The mean and standard deviation were recorded about continuous data, while the events’ number and participants’ number were noted about categorical data. Disagreements were resolved by another researcher.

### 2.6 Quality assessment

Two researchers assessed the literature quality using the Cochrane Risk of Bias tool. Seven components including random sequence generation, allocation concealment, blinding, completeness of outcome data, reporting bias, and other biases. Studies were classified as “high risk,” “low risk,” or “unclear risk.” Disagreements were resolved by another researcher.

### 2.7 Statistical analysis

EndNote 9.0, Excel, RevMan 5.4, and Stata 15.1 were used for literature management, data organization, and analysis. For binary outcomes, the risk ratio (RR) was used; for continuous outcomes, the standardized mean difference (SMD) was applied. Cochrane’s Q test and the *I* ^2^ test were used for heterogeneity assessment, with significant heterogeneity defined by p ≥ 0.1 and *I* ^2^ < 50%. A random-effects model was applied with α = 0.05. Sensitivity analysis was performed using the one-by-one elimination method, and funnel plots along with Egger’s test were used to assess publication bias. Subgroup analysis, based on population, intervention duration, and age, was performed to assess result stability and identify potential sources of heterogeneity.

## 3 Results

### 3.1 Study searching and selection

**FIGURE 1:**
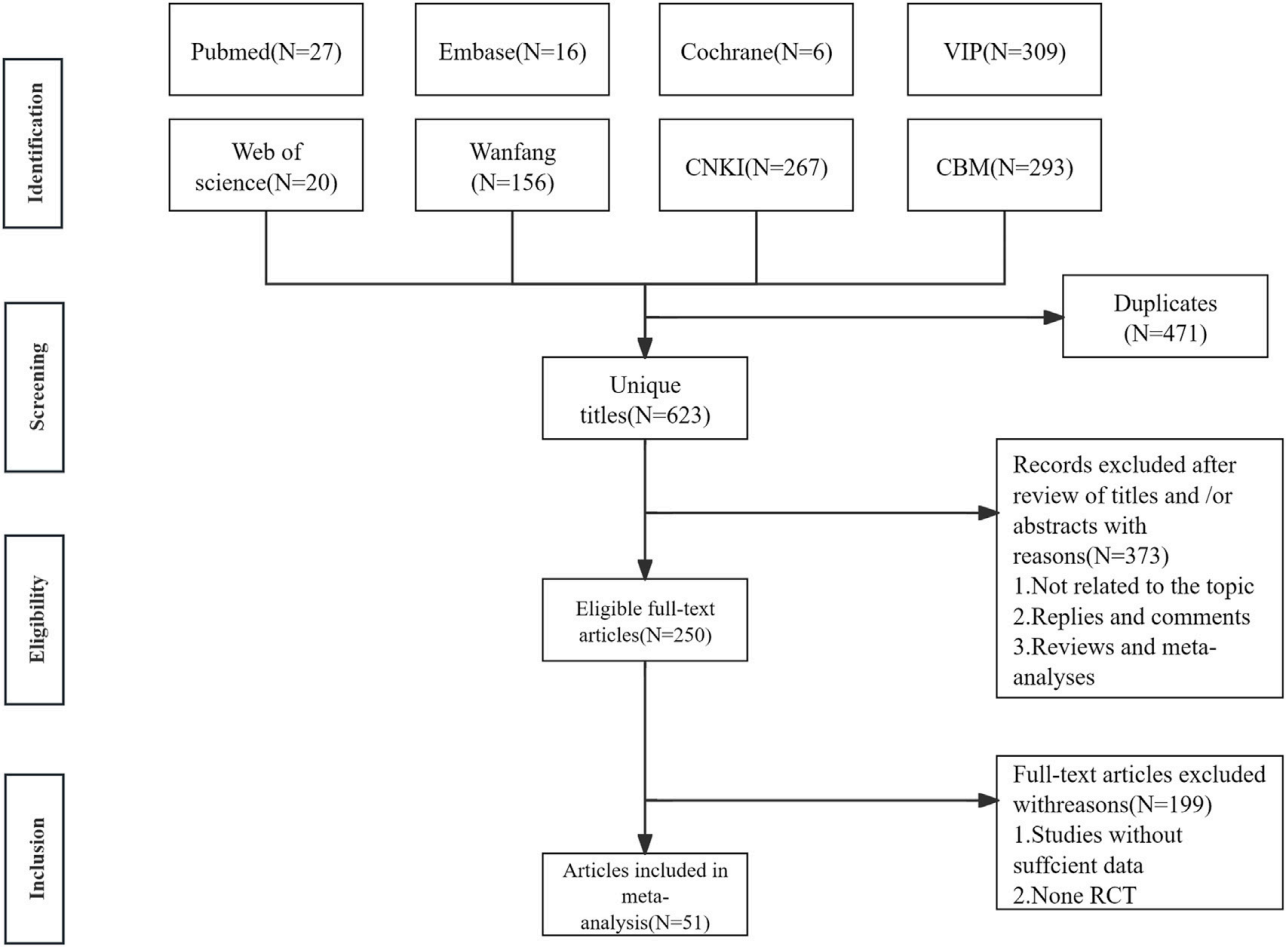
The literature searching and screening flowchart.

1904 articles with 623 remaining after excluding 471 duplicates included. 373 literatures were excluded according to the title and abstract. After reviewing the full texts, 199 articles were excluded, leaving 51 articles, as shown in Figure 1.

### 3.2 Study characteristics

Fifty-one RCTs’ studies (Wen et al., 2013; Cui et al., 2008; Sun et al., 2000; Luo et al., 2002; Liu et al., 2003; Wang et al., 2004; Wang et al., 2005; Duan, 2006; Wang et al., 2006; Wang; Huang et al., 2007; Lui et al., 2007; Lv, 2008; Ma, 2008; Wang, 2008; Yan et al., 2008; Yang, 2008; Sun et al., 2009; Yang et al., 2009; Chu, 2010; Kong et al., 2010; Wei et al., 2010; Wu et al., 2010; Yu et al., 2010; Su et al., 2011; Liu and LI, 2012; Lan et al., 2013; Cao et al., 2014; Guo et al., 2014; Ruan and Ruan, 2014; Wang, 2014; Wang and Gai, 2014; Xue et al., 2014; Di, 2015; Huang, 2015; Tang and Yu, 2015; Wang, 2015; Wang et al., 2015; Wu et al., 2010; Wang et al., 2016; Zhou et al., 2016; Bai et al., 2017; Mi et al., 2017; Xiao, 2017; Qin et al., 2018; Chen et al., 2021; Li P.-X. et al., 2021; Wu et al., 2021; Deng and Jia, 2024; Dong et al., 2024) with 9,577 patients were included, 5,019 in the intervention group and 4,558 in the control group, both with sample sizes exceeding 100. The average age ranged from 51.20 to 72.67 years in the intervention group and from 51.20 to 71.83 years in the control group. Treatment duration varied from 2 to 48 weeks. The control group received conventional treatments such as simvastatin, aspirin, liraglutide, edaravone, and acupuncture. The intervention group received Tongxinluo capsules in addition to the control treatments (Table 1).

**TABLE 1:** The basic characteristics of literature.

Study	study period	region	study design	Population	Intervention	Intervention time	Control
Liu et al. (2003)	2002–2003	China	RCT	Cerebral infarction	Tongxinluo	30 days	Buyang also five soup + acupuncture
Xiao (2017)	2013–2015	China	RCT	Acute cerebral infarction	Tongxinluo + Conventional treatment + acupuncture	—	Conventional treatment
Deng and Jia (2024)	2021–2023	China	RCT	Cerebral infarction + Type 2 diabetes	Tongxinluo + Conventional treatment	12 weeks	Conventional treatment + liraglutide
Deng and Jia (2024)	2021–2024	China	RCT	Cerebral infarction + Type 2 diabetes	Tongxinluo + Conventional treatment + liraglutide	12 weeks	Conventional treatment + liraglutide
Cao et al. (2014)	—	China	RCT	Cerebral infarction	Tongxinluo	28 days	Naoxintong capsule
Su et al. (2011)	2003–2004	China	RCT	Cerebral infarction	Tongxinluo	28 days	Naoxintong capsule
Wu et al. (2010)	2008–2009	China	RCT	Acute cerebral infarction	Tongxinluo + Conventional treatment	—	Simvastatin + Conventional treatment
Wang et al. (2004)	—	China	RCT	Acute cerebral infarction	Tongxinluo + Conventional treatment	3 weeks	Cerebral thrombosis tablet + Conventional treatment
Yan et al. (2008)	2003–2004	China	RCT	Acute cerebral infarction	Tongxinluo + Conventional treatment	12 months	Conventional treatment
Yao et al. (2006)	—	China	RCT	Cerebral infarction	Tongxinluo + aspirin	15 days	Dihydroergosine mesylate + aspirin
Wu and Li (2015)	2009–2013	China	RCT	Cerebral infarction + Type 2 diabetes	Tongxinluo	4 weeks	Simvastatin + Conventional treatment
Wu and Li (2015)	2,209–2013	China	RCT	Cerebral infarction + Type 2 diabetes	Tongxinluo + Simvastatin	4 weeks	Simvastatin + Conventional treatment
Huang (2015)	2008–2011	China	RCT	Cerebral infarction + Type 2 diabetes	Tongxinluo	4 weeks	Simvastatin + Conventional treatment
Huang (2015)	2008–2011	China	RCT	Cerebral infarction + Type 2 diabetes	Tongxinluo + Simvastatin	4 weeks	Simvastatin + Conventional treatment
Kong et al. (2010)	2004–2009	China	RCT	Acute cerebral infarction	Tongxinluo + Conventional treatment	3 weeks	Conventional treatment
Lui et al. (2007)	2002–2005	China	RCT	Cerebral infarction convalescence period	Tongxinluo + Conventional treatment	30 days	Conventional treatment
Wang (2006)	2002–2005	China	RCT	Cerebral infarction convalescence period	Tongxinluo + Conventional treatment	24 weeks	Conventional treatment
Lan et al. (2013)	2011–2013	China	RCT	Acute cerebral infarction	Tongxinluo + Conventional treatment + Acupuncture and moxibustion	30 days	Conventional treatment + acupuncture
Guo et al. (2014)	2012–2013	China	RCT	Cerebral infarction	Tongxinluo	8 weeks	Simvastatin
Lv (2008)	2005–2006	China	RCT	Acute cerebral infarction	Tongxinluo	4 weeks	Conventional treatment
Xue et al. (2014)	—	China	RCT	Cerebral infarction	Tongxinluo + Atorvastatin + aspirin	3 months	atorvastatin + aspirin
Wang and Gai (2014)	2010–2012	China	RCT	Acute cerebral infarction	Tongxinluo + Clopidogrel + aspirin	14 days	aspirin
Li et al. (2021a)	2015–2020	China	RCT	Acute cerebral infarction	Tongxinluo + Clopidogrel	1 month	clopidogrel
Wang et al. (2016)	2014–2015	China	RCT	Cerebral infarction	Tongxinluo + Conventional treatment + Probucol	12 months	probucol + Conventional treatment
Zhou et al. (2016)	2014–2015	China	RCT	hypertensive intracerebral hemorrhage	Tongxinluo + Conventional treatment + Nimodipine	4 weeks	nimodipine + Conventional treatment
Chen et al. (2021)	2017–2019	China	RCT	Acute cerebral infarction	Tongxinluo + Conventional treatment + Edaravone	—	Edaravone + Conventional treatment
Wu et al. (2021)	2017–2019	China	RCT	Acute cerebral infarction	Tongxinluo + Conventional treatment + Edaravone	14 days	Edaravone + Conventional treatment
Ma (2008)	—	China	RCT	Cerebral infarction	Tongxinluo + Conventional treatment	1 month	Weinolutong + Conventional treatment
Wang and Gai (2014)	2011–2013	China	RCT	hypertensive cerebral hemorrhage	Tongxinluo + Conventional treatment	—	Conventional treatment
Wang (2006)	2002–2005	China	RCT	Acute cerebral infarction	Tongxinluo + Conventional treatment	2 weeks	Conventional treatment
Ruan and Ruan (2014)	2009–2013	China	RCT	Acute cerebral infarction	Tongxinluo + Salvia ligustrazine injection + biaspirin	15 days	Salvia ligustrazine injection + biaspirin
Wen et al. (2013)	2010–2013	China	RCT	Acute cerebral infarction	Tongxinluo + Conventional treatment	4 weeks	Conventional treatment
Wei et al. (2010)	2006–2006	China	RCT	Acute cerebral infarction	Tongxinluo + Conventional treatment	3 months	Conventional treatment
Liu and Li (2012)	2009–2011	China	RCT	Cerebral infarction	Tongxinluo + Conventional treatment	3 months	Conventional treatment
Di (2015)	—	China	RCT	Acute cerebral infarction	Tongxinluo + Conventional treatment	30 days	Conventional treatment
Chu (2010)	2006–2008	China	RCT	Cerebral infarction	Tongxinluo + aspirin	28 days	aspirin
Luo et al. (2002)	2001–2002	China	RCT	Acute cerebral infarction	Tongxinluo + Conventional treatment	4 weeks	Danshen injection + Conventional treatment
Cui et al. (2008)	2006–2007	China	RCT	Acute cerebral infarction	Tongxinluo + Conventional treatment + puerarin	30 days	Conventional treatment + puerarin
Tang and Wu (2015)	2011–2013	China	RCT	Cerebral infarction	Tongxinluo + Atorvastatin	6 eeksw	atorvastatin
Sun et al. (2000)	—	China	RCT	Cerebral infarction	Tongxinluo + Danshen injection	4 weeks	Danshen injection
Wang et al. (2005)	2003–2004	China	RCT	Cerebral infarction	Tongxinluo + Conventional treatment	14 days	Conventional treatment
Yu et al. (2010)	2007–2008	China	RCT	Cerebral infarction complicated with hyperlipidemia	Tongxinluo + Cytidiphosphate choline, aspirin, A-Lo	1 month	Cytidiphosphate choline, aspirin, A-Lo
Yang et al. (2008)	2007–2008	China	RCT	Acute cerebral infarction	Tongxinluo + Cytidiphosphate choline, aspirin, Salvia miltiorrhiza	4 weeks	Cytidiphosphate choline, aspirin, Salvia miltiorrhiza
Wang (2015)	2010–2013	China	RCT	Acute cerebral infarction	Tongxinluo + butylphenylpeptide	2.5 months	butylphenylpeptide
Yang et al. (2009)	2007–2009	China	RCT	Acute cerebral infarction	Tongxinluo + Cytidiphosphate choline, aspirin, Salvia miltiorrhiza, piracetam	4 weeks	Cytidiphosphate choline, aspirin, Salvia miltiorrhiza, piracetam
Mi et al. (2017)	2014–2015	China	RCT	Cerebral infarction	Tongxinluo + Conventional treatment	6 months	Conventional treatment
Duan (2006)	2004–2005	China	RCT	Acute cerebral infarction	Tongxinluo + Conventional treatment	4 weeks	metabolite Xueshuantong capsule + Conventional treatment
Huang et al. (2007)	2004–2005	China	RCT	Acute cerebral infarction	Tongxinluo + Conventional treatment	2 weeks	Conventional treatment
Wang et al. (2008)	—	China	RCT	Cerebral infarction	Tongxinluo + Cytidiphosphate choline	4 weeks	Cytidiphosphate choline + piracetam
Qin et al. (2018)	2016–2017	China	RCT	Cerebral infarction	Tongxinluo	—	acupuncture
Wang et al. (2015)	2012–2013	China	RCT	Cerebral infarction	Tongxinluo	14 days	acupuncture
Bai et al. (2017)	2012–2014	China	RCT	Cerebral infarction	Tongxinluo + Conventional treatment	12 months	Conventional treatment
Sun et al. (2009)	2007–2008	China	RCT	Acute cerebral infarction	Tongxinluo + Conventional treatment	8 weeks	Conventional treatment
Dong et al. (2024)	2014–2016	United States of America	RCT	Acute cerebral infarction	Tongxinluo	90 days	placebo

Study	Patients		Mean/median age		Male		Mean/median course of disease		Research findings
	Intervention	Control	Intervention	Control	Intervention	Control	Intervention	Control	
Liu et al. (2003)	60	60	57.26 ± 11.49	58.74 ± 11.74	91		17.22 ± 4.45 d	19.76 ± 6.02 d	Tongxinluo demonstrated an overall efficacy rate of 81.67% and a marked improvement rate of 48.33% in stroke patients. No significant adverse reactions were observed during or after the treatment period.
Xiao (2017)	63	63	65.1 ± 4.7	64.7 ± 4.1	35	33	—		**Tongxinluo combined with acupuncture significantly improves the efficacy rate and NIHSS scores in stroke patients compared to conventional treatment.**
Deng and Jia (2024)	50	50	62.59 ± 6.20		91		—		Compared to liraglutide alone, Tongxinluo combined with liraglutide significantly improves patients’ IMT, TC, TG, LDL-C, TNF-α, IL-6, hs-CRP, and HDL-C levels, with no serious adverse reactions observed during the treatment period.
Deng and Jia (2024)	50	50							
Cao et al. (2014)	198	65	63.1 ± 9.4	63.3 ± 9.2	111	45	2.6 ± 2.3 m	2.3 ± 1.4 m	Compared to Naoxintong, Tongxinluo treatment significantly improves the efficacy rate and traditional Chinese medicine (TCM) syndrome scores in stroke patients, with no noticeable adverse reactions observed during the treatment period.
Su et al. (2011)	63	61	—	—	31	33	—	—	Tongxinluo treatment significantly improves the efficacy rate in stroke patients compared to Naoxintong.
Wu et al. (2010)	67	67	—	—	—	—	—	—	Compared to conventional treatment, Tongxinluo significantly improves TNF-α, hs-CRP, LDL-C, and TC levels in stroke patients.
Wang et al. (2004)	52	50	68.2 ± 3.3	67.4 ± 3.5	29	28	—	—	Compared to conventional treatment, Tongxinluo significantly improves clinical symptoms, neurological deficit scores, and plasma levels of endothelin (ET), calcitonin gene-related peptide (CGRP), and nitric oxide (NO) in stroke patients.
Yan et al. (2008)	182	178	—	—	—	—	—	—	**Compared to conventional treatment, Tongxinluo significantly improves Barthel index scores and neurological deficit scores in stroke patients, with no significant adverse reactions observed during the treatment period.**
Yao(2006)	228	38	—	—	108	17	—	—	Compared to conventional treatment, Tongxinluo significantly improves the efficacy rate in stroke patients.
Wu and Li (2015)	51	51	66.1 ± 10.5	65.7 ± 11.4	30	31	—	—	Compared to conventional treatment, the Tongxinluo group showed significant improvements in TC, TG, LDL-C, HDL-C, TNF-α, and hs-CRP levels in stroke patients.
Wu and Li (2015)	52	51	63.6 ± 10.7		35		—		
Huang (2015)	50	50	51.2 ± 5.1	51.2 ± 5.2	27	28	9.1 ± 1.1 h	9.3 ± 1.2 h	**Compared to simvastatin alone, Tongxinluo combined with simvastatin significantly improves TC, TG, LDL-C, HDL-C, and hs-CRP levels, as well as the overall efficacy rate in stroke patients.**
Huang (2015)	50		51.4 ± 5.3		27		9.4 ± 1.3 h		
Kong et al. (2010)	186	189	61.4 ± 11.3	61.3 ± 11.2	110	108	—	—	Compared to conventional treatment, Tongxinluo significantly improves clinical efficacy and hemorheological parameters in stroke patients.
Lui et al. (2007)	51	51	61.35 ± 12.61	62.93 ± 7.62	30	29	6.6 ± 0.78 m	6.5 ± 0.69 m	Compared to conventional treatment, Tongxinluo significantly improves neurological function, enhances activities of daily living, and increases overall clinical efficacy in stroke patients, with no serious adverse reactions observed during the treatment period.
Wang (2006)	65	59	—	—	—	—	—	—	Compared to conventional treatment, Tongxinluo significantly improves motor function as measured by the Fugl-Meyer (FM) assessment and cognitive function as assessed by the Mini-Mental State Examination (MMSE), enhances limb function recovery, and to some extent improves hemorheological parameters in stroke patients.
Lan et al. (2013)	60	60	57.0 ± 2.3	56.0 ± 3.6	36	38	29.2 ± 9.6 h	28.0 ± 12.6 h	**Compared to conventional treatment, Tongxinluo significantly improves the efficacy rate, neurological deficit scores, Barthel index scores, levels of hs-CRP, TNF-α, IL-6, blood lipids, and hemorheological parameters in stroke patients, with no serious adverse reactions observed during the treatment period.**
Guo et al. (2014)	84	84	66.4士7.8	65.9 ± 8.7	45	47	—	—	Compared to simvastatin alone, Tongxinluo combined with simvastatin significantly improves total cholesterol (TC), triglycerides (TG), high-density lipoprotein cholesterol (HDL-C), low-density lipoprotein cholesterol (LDL-C), apolipoprotein A1 (ApoA1), apolipoprotein B (ApoB), and the ApoA1/ApoB ratio in stroke patients, indicating an improvement in dyslipidemia. No noticeable adverse reactions were observed during the treatment period.
Lv (2008)	60	60	60 ± 10	61 ± 10	39	33	—	—	Compared to conventional treatment, Tongxinluo significantly improves the efficacy rate, degree of neurological deficits, quality of life scores, and lipid profiles in stroke patients.
Xue et al. (2014)	74	74	66.1 ± 7.2	65.8 ± 6.3	42	43	—	—	Compared to conventional treatment alone, Tongxinluo combined with conventional therapy significantly improves total cholesterol, triglycerides, low-density lipoprotein cholesterol, C-reactive protein, interleukin-6, vascular stenosis rate, platelet aggregation rate, and high-density lipoprotein cholesterol in stroke patients, with no noticeable adverse reactions observed during the treatment period.
Wang (2014)	120	80	51.3 ± 10.1	53.0 ± 9.8	80	51	—	—	Compared to conventional treatment, Tongxinluo combined with clopidogrel and aspirin significantly improves neurological deficit scores in stroke patients, with no serious adverse reactions observed during the treatment period.
Li et al. (2021b)	62	61	53.7 ± 8.5	54.6 ± 8.4	29	30	24.6 ± 6.0 h	23.9 ± 5.8 h	Compared to conventional treatment combined with clopidogrel, Tongxinluo combined with clopidogrel significantly improves the overall clinical efficacy, cognitive function during the recovery phase, and quality of life in stroke patients.
Wang et al. (2016)	53	53	62.5 ± 9.8	63.8 ± 9.4	33	31	—	—	**Compared to probucol alone, Tongxinluo combined with probucol significantly reduces serum levels of ox-LDL, MMP-7, and IL-18 in stroke patients, suggesting a potential role in delaying or reversing the progression of carotid atherosclerotic plaques. No significant adverse reactions were observed during the treatment period.**
Zhou et al. (2016)	51	51	56.9 ± 6.6	55.6 ± 7.6	32	28	9.8 ± 3.5 y	10.1 ± 4.2 y	Compared to nimodipine alone, Tongxinluo combined with nimodipine significantly reduces serum levels of hs-CRP and Tau protein, increases IGF-1 levels, and improves neurological deficit symptoms in stroke patients, with no serious adverse reactions observed during the treatment period.
Chen et al. (2021)	57	57	72.67 ± 7.43	71.83 ± 7.51	32	31	9.85 ± 2.69 h	9.67 ± 2.84 h	Compared to edaravone alone, Tongxinluo combined with edaravone significantly improves neurological deficit scores (NIHSS), serum total cholesterol (TC), triglycerides (TG), low-density lipoprotein cholesterol (LDL-C), high-sensitivity C-reactive protein (hs-CRP), nitric oxide (NO) levels, plaque area, and intima-media thickness (IMT) in stroke patients.
Wu et al. (2021)	53	53	61.41 ± 8.12	60.94 ± 8.21	29	32	—	—	Compared to edaravone alone, Tongxinluo combined with edaravone significantly improves lipid profiles and inflammatory markers, enhances the overall treatment efficacy, and shows no significant adverse reactions during the treatment period in stroke patients.
Ma (2008)	56	56	—	—	35	36	—	—	**Compared to Vinpocetine Capsules combined with conventional treatment, Tongxinluo combined with conventional treatment significantly improves overall efficacy, limb function recovery, and cognitive function scores in stroke patients.**
Wang (2014)	56	54	—	—	—	—	—	—	Compared to conventional treatment alone, Tongxinluo combined with conventional therapy significantly improves neurological function and blood viscosity in stroke patients.
Wang (2006)	56	56	62.31 ± 10.05	61.64 ± 9.87	34	32	—	—	Compared to conventional treatment alone, Tongxinluo combined with conventional therapy significantly improves the overall efficacy rate and neurological function in stroke patients, with no noticeable adverse reactions observed during the treatment period.
Ruan et al. (2014)	63	63	61.5 ± 7.2	61.1 ± 6.9	39	38	—	—	**Compared to conventional treatment alone, Tongxinluo combined with conventional therapy significantly improves the overall efficacy rate, neurological function, and hemorheological parameters in stroke patients.**
Wen et al. (2013)	136	142	64.9	63.8	64	70	—	—	**Compared to conventional treatment alone, Tongxinluo combined with conventional therapy significantly improves the overall efficacy rate, neurological deficit scores, and Barthel index in stroke patients, with no noticeable adverse reactions observed during the treatment period.**
Wei et al. (2010)	50	50	68.64 ± 10.52	69.05 ± 10.53	23	25	—	—	Compared to conventional treatment alone, Tongxinluo combined with conventional therapy significantly improves total cholesterol (TC), triglycerides (TG), blood glucose levels, activities of daily living (ADL), and modified Rankin Scale (mRS) scores in stroke patients.
Liu et al. (2012)	50	56	—	—	—	—	—	—	**Compared to conventional treatment alone, Tongxinluo combined with conventional therapy significantly improves neurological deficit scores, lipid profiles, carotid plaque burden, and lumen stenosis, thereby enhancing overall clinical efficacy in stroke patients.**
Di (2015)	72	72	—	—	—	—	—	—	Compared to conventional treatment alone, Tongxinluo combined with conventional therapy significantly improves neurological deficit scores and blood viscosity in stroke patients, with no noticeable adverse reactions observed during the treatment period.
Chu (2010)	50	50	68.7	69.2	28	30	—	—	Compared to conventional treatment alone, Tongxinluo combined with conventional therapy significantly improves neurological deficit scores and increases the overall efficacy rate in stroke patients.
Luo et al. (2002)	88	72	62.76	61.48	58	48	—	—	Compared to Salvia miltiorrhiza injection combined with conventional treatment, Tongxinluo combined with conventional therapy significantly improves lipid metabolism, blood viscosity, hemorheological parameters, and platelet aggregation function in stroke patients. It effectively enhances cerebral blood circulation, reduces delayed neuronal injury, and improves neurological deficits.
Cui et al. (2008)	72	40	63.2 ± 12.1	59.6 ± 15.9	43	23	—	—	Compared to conventional treatment alone, Tongxinluo combined with conventional therapy significantly improves neurological deficit scores and increases the overall efficacy rate in stroke patients.
Tang et al. (2015)	60	60	55.6 ± 2.5	54.9 ± 3.0	27	28	—	—	**Compared to conventional treatment alone, Tongxinluo combined with conventional therapy significantly improves serum cytokine levels and enhances the overall clinical efficacy in stroke patients.**
Sun et al. (2000)	50	50	—	—	28	30	—	—	Compared to Salvia miltiorrhiza injection, Tongxinluo treatment significantly improves neurological deficits and enhances clinical efficacy in stroke patients.
Wang et al. (2005)	105	102	61	60	58	57	11 h	10 h	Compared to conventional treatment alone, Tongxinluo combined with conventional therapy significantly improves vertebrobasilar insufficiency and enhances clinical efficacy in stroke patients, with no serious adverse reactions observed during the treatment period.
Yu et al. (2010)	82	80	—	—	42	39	—	—	**Compared to conventional treatment alone, Tongxinluo combined with conventional therapy significantly improves lipid profiles in stroke patients.**
Yang (2008)	67	67	62 ± 7.35	61 ± 6.64	40	38	—	—	**Compared to conventional treatment alone, Tongxinluo combined with low-molecular-weight heparin and conventional therapy significantly improves overall therapeutic efficacy and neurological deficits in stroke patients.**
Wang (2015)	60	60	64.46 ± 7.24	63.24 ± 7.36	37	35	—	—	**Compared to butylphthalide alone, Tongxinluo combined with butylphthalide significantly improves overall therapeutic efficacy, blood viscosity, and neurological deficits in stroke patients, with no noticeable adverse reactions observed during the treatment period.**
Yang et al. (2009)	128	128	63 ± 6.41	62.8	70	75	—	—	**Compared to conventional treatment alone, Tongxinluo combined with low-molecular-weight heparin significantly improves overall therapeutic efficacy and neurological deficits in stroke patients.**
Mi et al. (2017)	84	84	58.6 ± 3.2	59.1 ± 2.7	56	55	7.4 ± 1.5 d	7.9 ± 1.1 d	Compared to conventional treatment alone, Tongxinluo combined with conventional therapy significantly improves carotid intima-media thickness, atherosclerotic plaque area, carotid stenosis rate, and reduces levels of hs-CRP and D-dimer in stroke patients.
Duan (2006)	66	64	60.1	60	34	34	—	—	Compared to conventional treatment alone, Tongxinluo combined with conventional therapy significantly improves overall therapeutic efficacy and hemorheological parameters in stroke patients, with no noticeable adverse reactions observed during the treatment period.
Huang et al. (2007)	95	97	59.3	57.6	53	57	—	—	Compared to conventional treatment alone, Tongxinluo combined with conventional therapy significantly improves overall therapeutic efficacy in stroke patients, with marked improvements in arrhythmia, ST-T changes, and myocardial enzyme levels. No noticeable adverse reactions were observed during the treatment period.
Wang (2008)	60	60	—	—	—	—	—	—	Compared to Naofukang combined with conventional treatment, Tongxinluo combined with conventional therapy significantly improves the efficacy rate, limb function recovery, and cognitive function scores in stroke patients.
Qin et al. (2018)	41	41	69.03 ± 5.17	68.14 ± 4.63	23	24	3.21 ± 0.92 m	3.19 ± 0.87 m	Compared to conventional treatment, Tongxinluo combined with acupuncture effectively promotes angiogenesis and neural repair, inhibits apoptosis, and enhances functional recovery in stroke patients.
Wang et al. (2015)	34	35	68 ± 10	67 ± 10	16	18	3.08 ± 1.52 m	3.28 ± 1.64 m	**Compared to conventional treatment, Tongxinluo combined with acupuncture improves bone marrow stem cell mobilization and neurological deficits in stroke patients.**
Bai et al. (2017)	68	77	66.88 ± 10.60	66.99 ± 10.24	32	33	—	—	Compared to conventional treatment alone, Tongxinluo combined with conventional therapy reduces the recurrence rate of stroke, promotes neurological recovery, and contributes to better blood pressure and lipid control, with no serious adverse reactions observed during the treatment period.
Sun et al. (2009)	105	103	62 ± 12	63 ± 13	56	53	—	—	Compared to conventional treatment alone, Tongxinluo combined with conventional therapy improves overall efficacy, neurological deficits, activities of daily living, and motor function in stroke patients.
Dong et al. (2024)	973	973	61.3	60.9	677	665	—	—	Compared to conventional treatment alone, Tongxinluo combined with conventional therapy improves the overall efficacy rate in stroke patients, with no serious adverse reactions observed during the treatment period.

### 3.3 Bias risk assessment results

**FIGURE 2:**
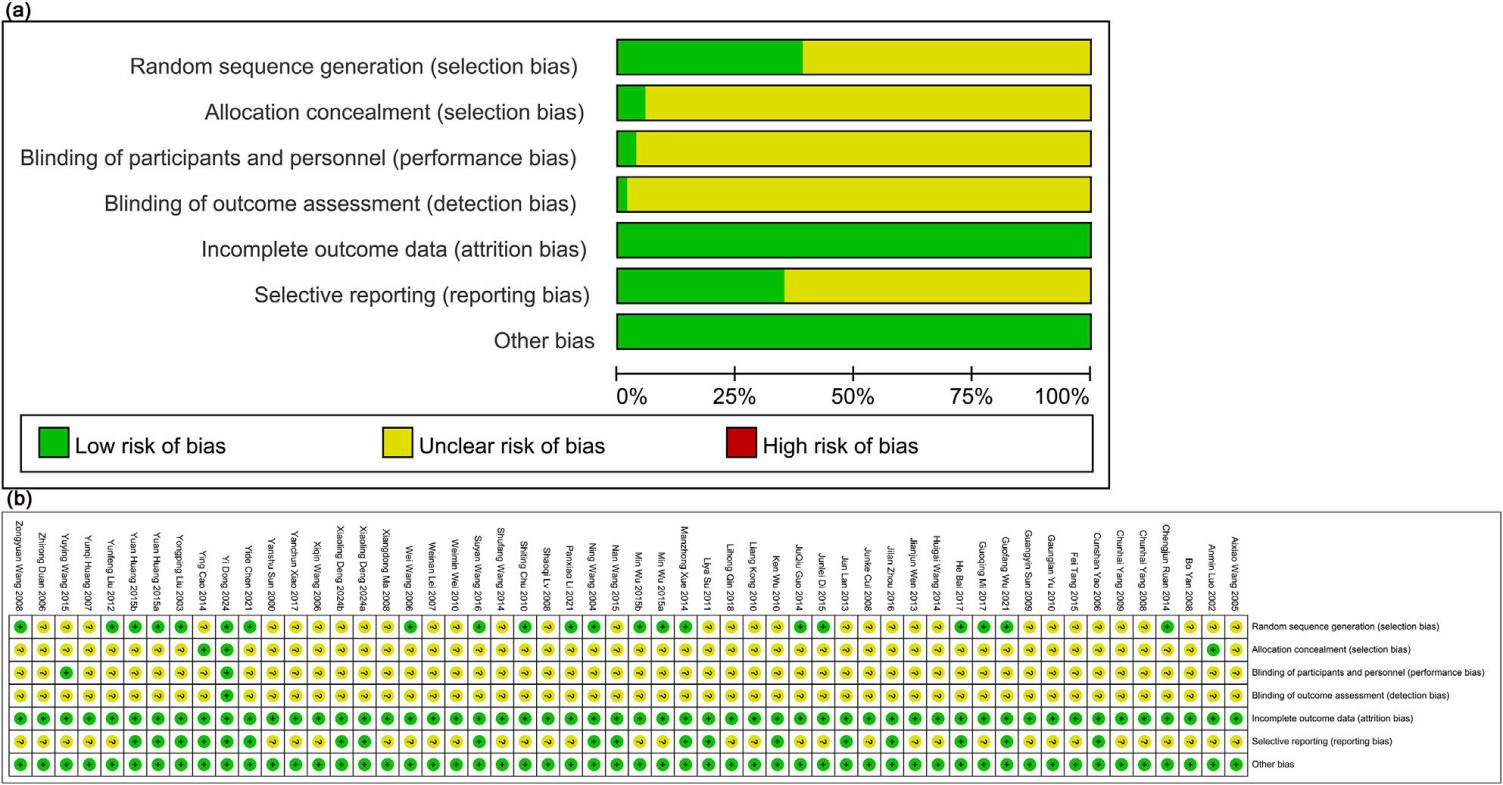
Literature quality evaluation. **(a)** Risk of bias graph; **(B)** Risk of bias summary.

The Cochrane risk assessment tool was performed to assess literature quality. Nineteen articles were classified as low risk based on the standard allocation method. Thirty-two studies did not specify the allocation method and were classified as having an unclear risk. Three studies employed a double-blind method for allocation concealment, classified as low risk, while the others did not provide this information and were classified as having an unclear risk. Two studies reported blinding, classified as low risk, while the others lacked details and were classified as having an unclear risk. All studies had complete outcome data, classified as low risk. Seventeen studies were classified as low risk for selective reporting, while the others were classified as unclear risk. No literature identified additional sources of bias, and these were classified as low risk (Figure 2).

### 3.4 Results of meta-analysis

#### 3.4.1 Overall efficacy

**FIGURE 3:**
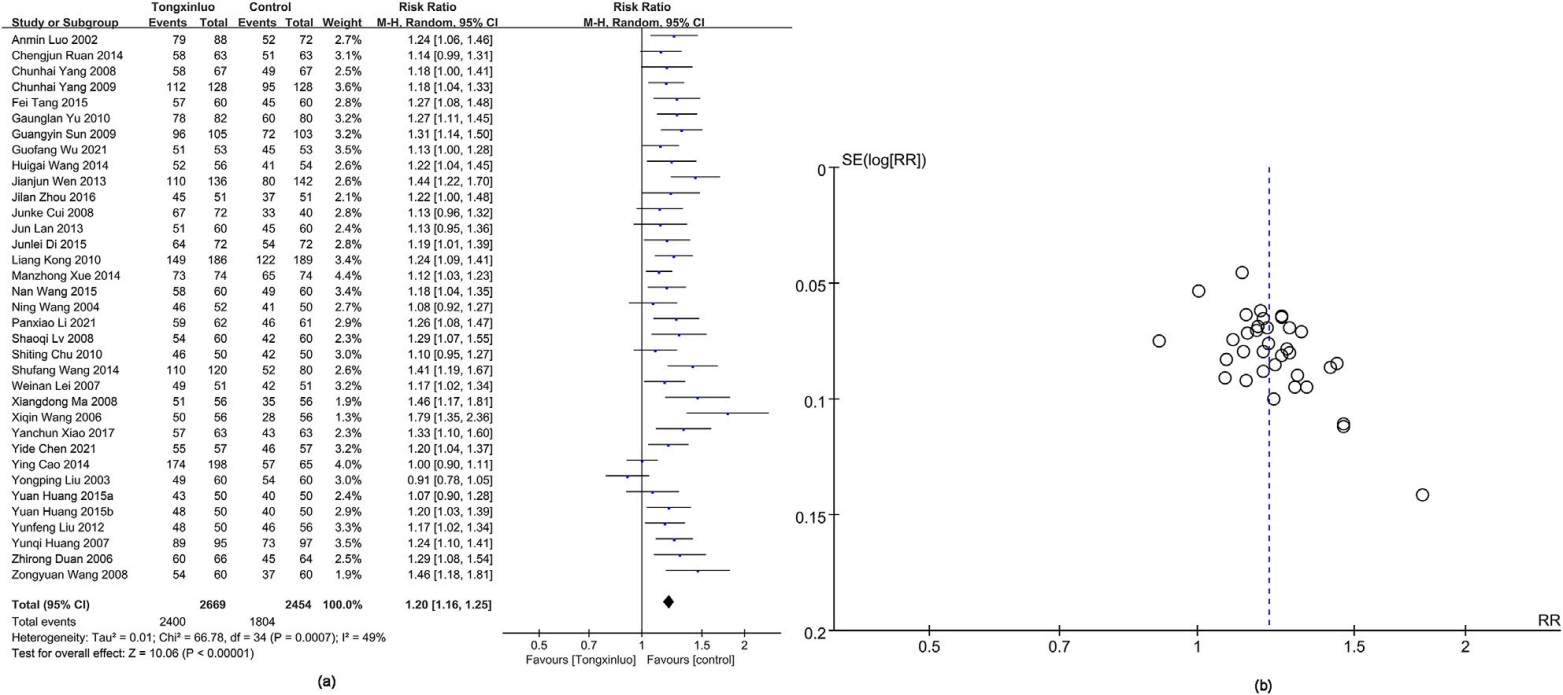
Forest and funnel map of overall efficacy. **(a)** Forest map; **(b)** Funnel map.

The overall efficacy was assessed in 35 studies (Wen et al., 2013; Cui et al., 2008; Luo et al., 2002; Liu et al., 2003; Wang et al., 2004; Wang; Huang et al., 2007; Lui et al., 2007; Lv, 2008; Ma, 2008; Yang, 2008; Sun et al., 2009; Yang et al., 2009; Chu, 2010; Kong et al., 2010; Yu et al., 2010; Liu and LI, 2012; Lan et al., 2013; Cao et al., 2014; Ruan and Ruan, 2014; Wang, 2014; Wang and Gai, 2014; Xue et al., 2014; Di, 2015; Huang, 2015; Tang and Yu, 2015; Wang, 2015; Zhou et al., 2016; Xiao, 2017; Chen et al., 2021; Li P.-X. et al., 2021; Wu et al., 2021). Results showed that Tongxinluo treatment for stroke was significantly more effective [RR = 1.20, 95% CI (1.16, 1.25)], with considerable heterogeneity (*I* ^2^ = 49%) (Figure 3a). Funnel plot analysis (Figure 3b) and Egger’s test (P = 0.001) indicated a significant publication bias.

#### 3.4.2 Adverse events

**FIGURE 4:**
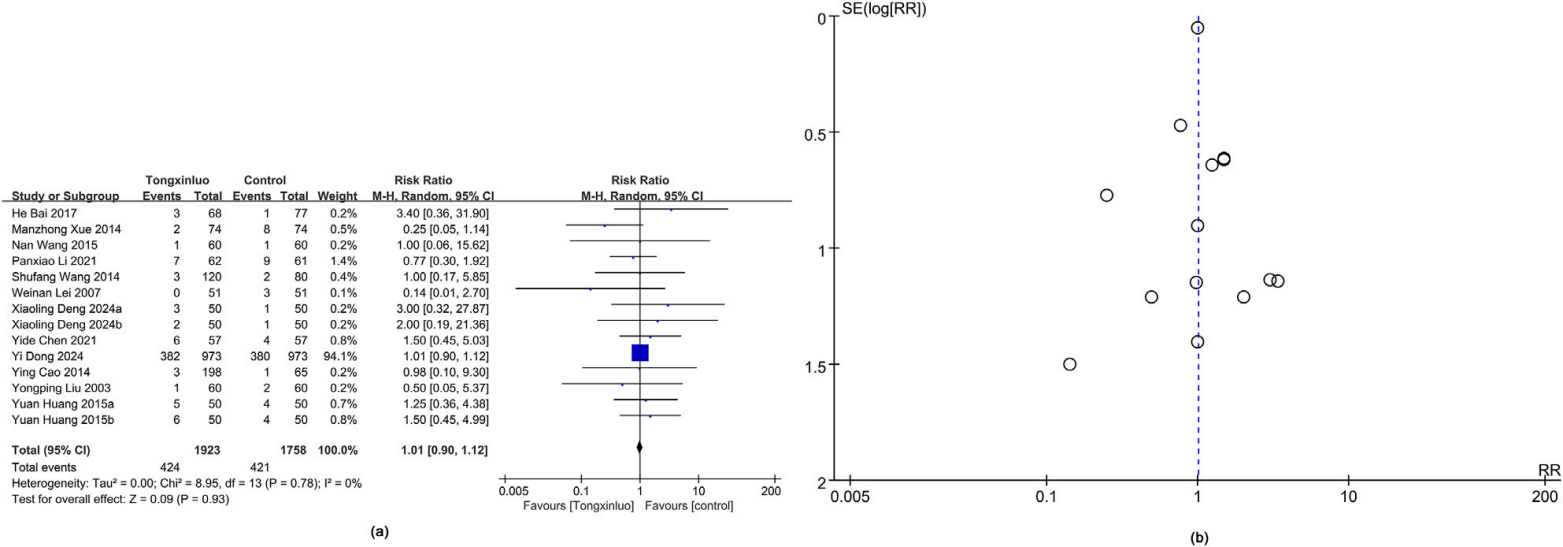
Forest and funnel map of adverse events. **(a)** Forest map; **(b)** Funnel map.

Adverse events were depicted in 14 studies (Liu et al., 2003; Lui et al., 2007; Cao et al., 2014; Wang and Gai, 2014; Xue et al., 2014; Huang, 2015; Wang, 2015; Bai et al., 2017; Chen et al., 2021; Li P.-X. et al., 2021; Deng and Jia, 2024; Dong et al., 2024). Results showed a higher adverse reaction rate in the Tongxinluo group (RR = 1.01), but the 95% CI (0.90, 1.12) indicated no significant difference, suggesting similar rates. Heterogeneity was 0% (*I* ^2^), showing no variability between groups (Figure 4a). Funnel plot analysis (Figure 4b) and Egger’s test (P = 0.988) revealed no publication bias.

#### 3.4.3 NIHSS score

**FIGURE 5:**
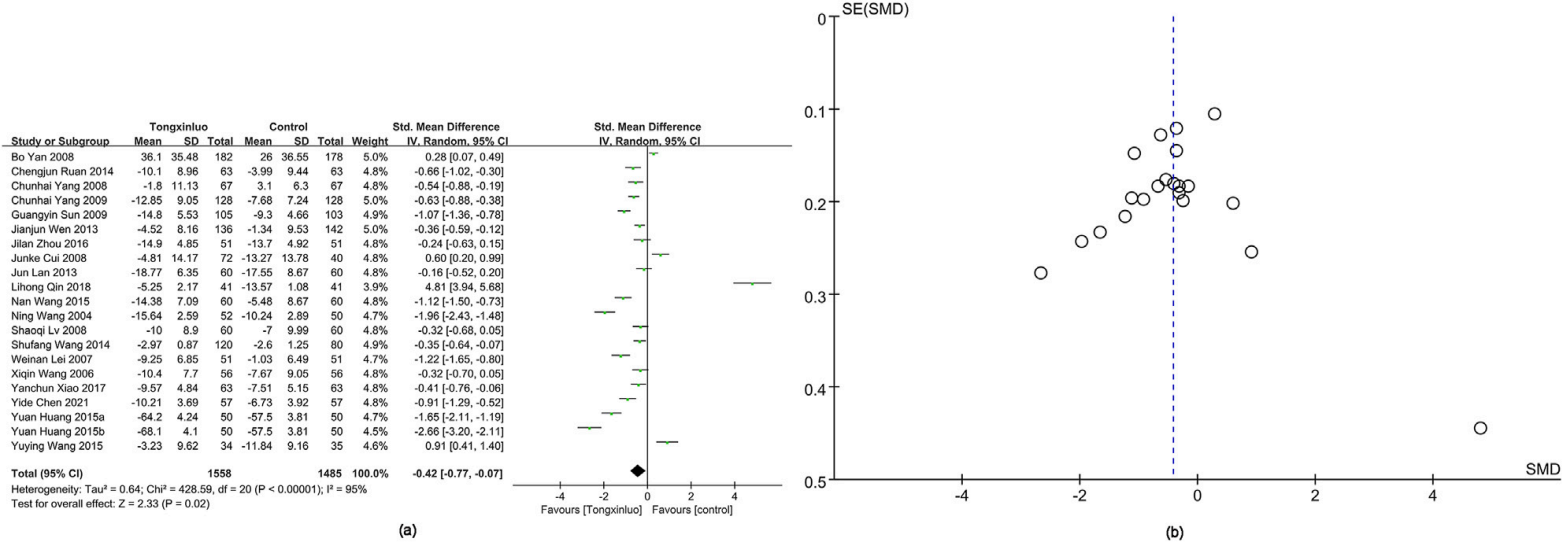
Forest and funnel map of NIHSS score. **(a)** Forest map; **(b)** Funnel map.

The NIHSS score was reported in 21 studies (Wen et al., 2013; Cui et al., 2008; Wang et al., 2004; Wang; Lui et al., 2007; Lv, 2008; Yan et al., 2008; Yang, 2008; Sun et al., 2009; Yang et al., 2009; Lan et al., 2013; Ruan and Ruan, 2014; Wang and Gai, 2014; Huang, 2015; Wang, 2015; Wang et al., 2015; Zhou et al., 2016; Xiao, 2017; Qin et al., 2018; Chen et al., 2021). Results showed a significantly greater improvement in the Tongxinluo group [SMD = −0.42, 95% CI (−0.77 to −0.07)], with no significant heterogeneity (*I* ^2^ = 95%) (Figure 5a). The funnel plot (Figure 5b) and Egger’s test (P = 0.845) indicated no publication bias.

#### 3.4.4 TC

**FIGURE 6:**
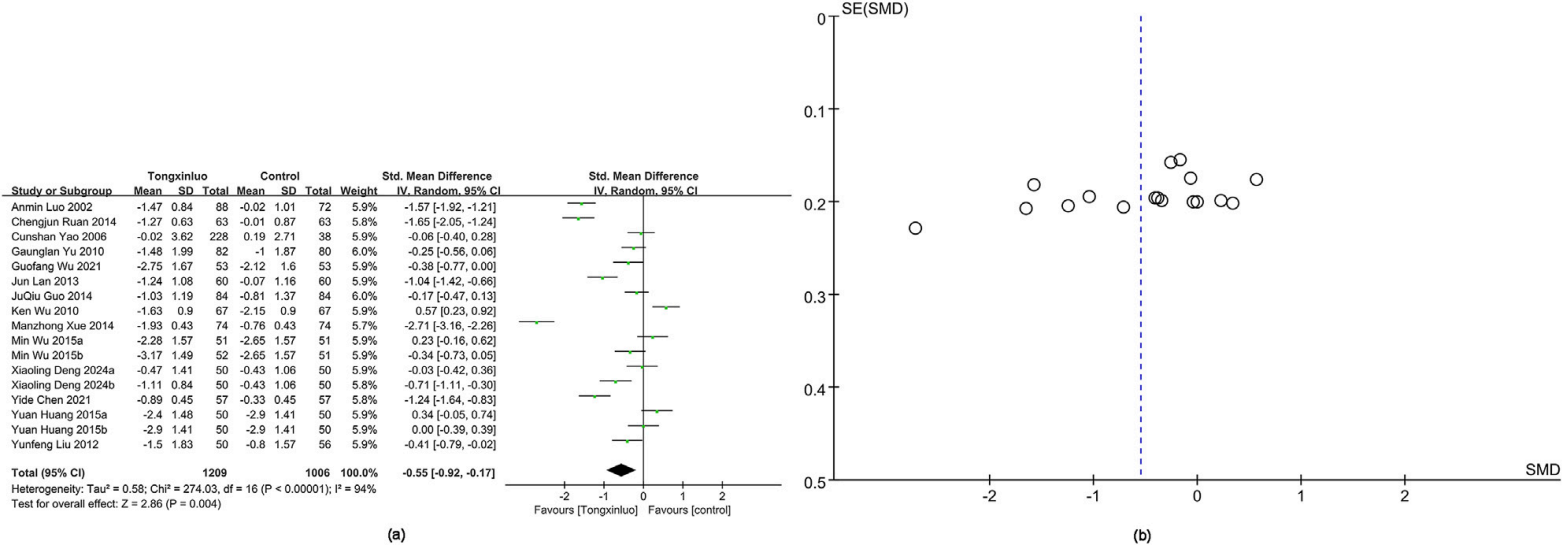
Forest and funnel map of TC. **(a)** Forest map; **(B)** Funnel map.

TC data were obtained from 17 studies (Luo et al., 2002; Yao et al.; Wu et al., 2010; Yu et al., 2010; Liu and LI, 2012; Lan et al., 2013; Guo et al., 2014; Ruan and Ruan, 2014; Xue et al., 2014; Huang, 2015; Wu and Li, 2015; Chen et al., 2021; Wu et al., 2021; Deng and Jia, 2024). The Tongxinluo group showed significantly better TC levels [SMD = −0.55, 95% CI (−0.92 to −0.17)], with no significant heterogeneity (*I* ^2^ = 94%) (Figure 6a). The funnel plot (Figure 6b) and Egger’s test (P = 0.075) indicated no publication bias.

#### 3.4.5 TG

TG data were obtained from 17 studies (Luo et al., 2002; Yao et al.; Wu et al., 2010; Yu et al., 2010; Liu and LI, 2012; Lan et al., 2013; Guo et al., 2014; Ruan and Ruan, 2014; Xue et al., 2014; Huang, 2015; Wu and Li, 2015; Chen et al., 2021; Wu et al., 2021; Deng and Jia, 2024). No significant difference in TG level improvement was observed [SMD = −0.22, 95% CI (−0.61 to −0.16)], with no significant heterogeneity (*I* ^2^ = 94%) (Supplementary Figure S1a). The funnel plot (Supplementary Figure S1b) and Egger’s test (P = 0.055) indicated no publication bias.

#### 3.4.6 hs-CRP

**FIGURE 7:**
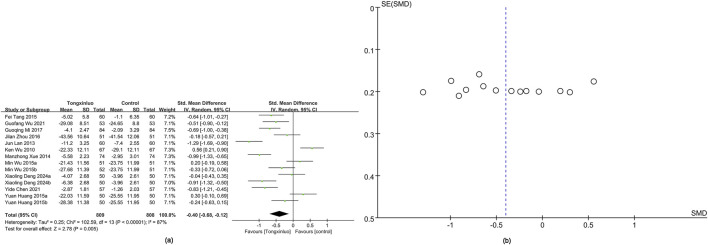
Forest and funnel map of hs-CRP. **(a)** Forest map; **(b)** Funnel map.

hs-CRP data were obtained from 14 studies (Wu et al., 2010; Lan et al., 2013; Xue et al., 2014; Huang, 2015; Tang and Yu, 2015; Wu and Li, 2015; Zhou et al., 2016; Mi et al., 2017; Chen et al., 2021; Wu et al., 2021; Deng and Jia, 2024). Results showed significantly greater hs-CRP improvement in the Tongxinluo group (SMD = −0.40, 95% CI [−0.68 to −0.12]), with no significant heterogeneity (*I* ^2^ = 87%) (Figure 7a). The funnel plot (Figure 7b) and Egger’s test (P = 0.816) indicated no publication bias.

#### 3.4.7 LDL

LDL data were obtained from 14 studies (Yao et al.; Wu et al., 2010; Yu et al., 2010; Liu and LI, 2012; Lan et al., 2013; Guo et al., 2014; Xue et al., 2014; Huang, 2015; Wu and Li, 2015; Chen et al., 2021; Deng and Jia, 2024). Results showed significantly greater LDL improvement in the Tongxinluo group (SMD = −0.84, 95% CI [−1.37 to −0.30]), with no significant heterogeneity (*I* ^2^ = 96%) (Supplementary Figure S2a). The funnel plot (Supplementary Figure S2b) and Egger’s test (P = 0.001) indicated publication bias.

#### 3.4.8 HDL

HDL data were obtained from 13 studies (Luo et al., 2002; Yao et al.; Wu et al., 2010; Yu et al., 2010; Lan et al., 2013; Guo et al., 2014; Xue et al., 2014; Huang, 2015; Wu and Li, 2015; Deng and Jia, 2024). Results showed significantly greater HDL improvement in the Tongxinluo group (SMD = 0.79, 95% CI [0.31, 1.28]), with no significant heterogeneity (*I* ^2^ = 95%) (Supplementary Figure S3a). The funnel plot (Supplementary Figure S3b) and Egger’s test (P = 0.017) indicated publication bias.

#### 3.4.9 TNF-α

TNF-α data were obtained from nine studies (Wu et al., 2010; Lan et al., 2013; Huang, 2015; Wu and Li, 2015; Wu et al., 2021; Deng and Jia, 2024). Results showed significantly greater TNF-α improvement in the Tongxinluo group (SMD = −0.60, 95% CI [−1.15 to −0.04]), with no significant heterogeneity (*I* ^2^ = 94%) (Supplementary Figure S4b). The funnel plot (Supplementary Figure S4b) and Egger’s test (P = 0.014) indicated publication bias.

#### 3.4.10 IL-6

IL-6 data were obtained from 10 studies (Lan et al., 2013; Xue et al., 2014; Huang, 2015; Tang and Yu, 2015; Wu and Li, 2015; Wu et al., 2021; Deng and Jia, 2024). Results showed significantly greater IL-6 improvement in the Tongxinluo group (SMD = −0.49, 95% CI [−0.80 to −0.18]), with no significant heterogeneity (*I* ^2^ = 85%) (Supplementary Figure S5a). The funnel plot (Supplementary Figure S5b) and Egger’s test (P = 0.986) indicated no publication bias.

#### 3.4.11 High-shear whole blood viscosity

High-shear whole blood viscosity data were obtained from five studies (Luo et al., 2002; Kong et al., 2010; Lan et al., 2013; Di, 2015; Wang, 2015). Results showed significantly greater improvement in the Tongxinluo group (SMD = −1.88, 95% CI [−2.87 to −0.88]), with no significant heterogeneity (*I* ^2^ = 97%) (Supplementary Figure S6a). The funnel plot (Supplementary Figure S6b) and Egger’s test (P = 0.092) indicated no publication bias.

#### 3.4.12 Low-shear whole blood viscosity

Low-shear whole blood viscosity data were obtained from five studies (Luo et al., 2002; Kong et al., 2010; Lan et al., 2013; Di, 2015; Wang, 2015). Results showed significantly greater improvement in the Tongxinluo group (SMD = −0.61, 95% CI [−0.94 to −0.28]), with no significant heterogeneity (*I* ^2^ = 82%) (Supplementary Figure S7a). The funnel plot (Supplementary Figure S7b) and Egger’s test (P = 0.391) indicated no publication bias.

#### 3.4.13 Barthel index score

Barthel Index score data were obtained from four studies (Wen et al., 2013; Lui et al., 2007; Yan et al., 2008; Sun et al., 2009). Results showed significantly greater improvement in the Tongxinluo group (SMD = 0.73, 95% CI [0.25, 1.20]), with no significant heterogeneity (*I* ^2^ = 92%) (Supplementary Figure S8a). The funnel plot (Supplementary Figure S8b) and Egger’s test (P = 0.501) indicated no publication bias.

#### 3.4.14 Plaque area

Plaque area data were obtained from four studies (Liu and LI, 2012; Wang et al., 2016; Mi et al., 2017; Chen et al., 2021). Results showed significantly greater improvement in the Tongxinluo group (SMD = −1.28, 95% CI [−1.84 to −0.71]), with no significant heterogeneity (*I* ^2^ = 88%) (Supplementary Figure S9a). The funnel plot (Supplementary Figure S9b) and Egger’s test (P = 0.616) indicated no publication bias.

#### 3.4.15 MMSE score

MMSE score data were obtained from four studies (Wang, 2006; Ma, 2008; Wang, 2008; Li P.-X. et al., 2021). Results showed significantly greater improvement in MMSE scores in the Tongxinluo group (SMD = 0.93, 95% CI [0.26, 1.61]), with no significant heterogeneity (*I* ^2^ = 92%) (Supplementary Figure S10a). The funnel plot (Supplementary Figure S10b) and Egger’s test (P = 0.049) indicated publication bias.

#### 3.4.16 FM score

FM score data were derived from three studies (Wang, 2006; Ma, 2008; Wang, 2008). Results showed significantly greater improvement in FM scores in the Tongxinluo group (SMD = 0.42, 95% CI [0.13, 0.71]), with significant heterogeneity (*I* ^2^ = 47%) (Supplementary Figure S11a). The funnel plot (Supplementary Figure S11b) and Egger’s test (P = 0.370) indicated no publication bias.

#### 3.4.17 Plasma viscosity

Plasma viscosity data were derived from seven studies (Luo et al., 2002; Duan, 2006; Kong et al., 2010; Lan et al., 2013; Ruan and Ruan, 2014; Wang, 2014; Di, 2015). Results showed significantly greater improvement in plasma viscosity in the Tongxinluo group (SMD = −1.28, 95% CI [−2.01 to −0.55]), with no significant heterogeneity (*I* ^2^ = 97%) (Supplementary Figure S12a). The funnel plot (Supplementary Figure S12b) and Egger’s test (P = 0.738) indicated no publication bias.

#### 3.4.18 Plasma fibrinogen

Plasma fibrinogen data were derived from six studies (Luo et al., 2002; Duan, 2006; Kong et al., 2010; Lan et al., 2013; Ruan and Ruan, 2014; Wang, 2015). Results showed significantly greater improvement in plasma fibrinogen in the Tongxinluo group (SMD = −0.47, 95% CI [−0.68 to −0.27]), with no significant heterogeneity (*I* ^2^ = 60%) (Supplementary Figure S13a). The funnel plot (Supplementary Figure S13b) and Egger’s test (P = 0.527) indicated no publication bias.

#### 3.4.19 Hematocrit

Hematocrit data were derived from two studies (Luo et al., 2002; Duan, 2006). Results showed significantly greater improvement in hematocrit in the Tongxinluo group (SMD = −1.26, 95% CI [−2.10 to −0.42]), with no significant heterogeneity (*I* ^2^ = 91%) (Supplementary Figure S14a), as shown in the funnel plot (Supplementary Figure S14b).

#### 3.4.20 NO

NO data were derived from two studies (Wang et al., 2004; Chen et al., 2021). Results showed significantly greater improvement in NO levels in the Tongxinluo group (SMD = 1.05, 95% CI [0.24, 1.86]), with no significant heterogeneity (*I* ^2^ = 87%) (Supplementary Figure S15a), as shown in the funnel plot (Supplementary Figure S15b).

#### 3.4.21 IL-18

IL-18 data were derived from two studies (Tang and Yu, 2015; Wang et al., 2016). Results showed no significant difference in IL-18 level improvement (SMD = −2.81, 95% CI [−6.25, 0.63]), with no significant heterogeneity (*I* ^2^ = 99%) (Supplementary Figure S16a), as indicated by the funnel plot (Supplementary Figure S16b).

#### 3.4.22 Platelet aggregation function

Platelet aggregation function data were derived from two studies (Luo et al., 2002; Xue et al., 2014). Results showed no significant difference in improvement (SMD = −0.74, 95% CI [−1.49, 0.01]), with no significant heterogeneity (*I* ^2^ = 86%) (Supplementary Figure S17a), as indicated by the funnel plot (Supplementary Figure S17b).

#### 3.4.23 Quality of life scores

Quality of life score data were derived from two studies (Lv, 2008; Li P.-X. et al., 2021). Results showed significantly greater improvement in the Tongxinluo group (SMD = 0.65, 95% CI [0.27, 1.03]), with no significant heterogeneity (*I* ^2^ = 54%) (Supplementary Figure S18a), as shown by the funnel plot (Supplementary Figure S18b).

### 3.5 Sensitivity analysis

A univariate sensitivity analysis was conducted to assess the total efficacy (Supplementary Figure S19a), adverse events (Supplementary Figure S19b), NIHSS scores (Supplementary Figure S19c), TC (Supplementary Figure S19d), TG (Supplementary Figure S19e), HDL (Supplementary Figure S19f), LDL (Supplementary Figure S19g), hs-CRP (Supplementary Figure S19h), TNF-α (Supplementary Figure S19i), IL-6 (Supplementary Figure S19j), high-shear whole blood viscosity (Supplementary Figure S19k), low-shear whole blood viscosity (Supplementary Figure S19l), Barthel scores (Supplementary Figure S19m), plaque area (Supplementary Figure S19n), FM scores (Supplementary Figure S19o), plasma viscosity (Supplementary Figure S19p), plasma fibrinogen (Supplementary Figure S19q), MMSE scores (Supplementary Figure S19r), red blood cell hematocrit, NO, IL-18, platelet aggregation function, and quality of life scores by sequentially excluding individual studies. This analysis assessed each study’s impact on the overall effect. Results showed significant changes in NIHSS scores, FM scores, MMSE scores, TG, and TNF-α after excluding certain studies, indicating instability in these outcomes. Caution is needed when interpreting results for these indicators. Excluding studies had minimal impact on the pooled effect size for other outcomes, indicating good stability.

### 3.6 Subgroup analysis

Subgroup analysis was conducted based on population, intervention duration, and disease duration to assess total efficacy and NIHSS scores. The total efficacy analysis indicated that Tongxinluo was effective across all subgroups. The NIHSS scores analysis showed that Tongxinluo was ineffective in studies with intervention durations longer than 4 weeks and in studies involving participants older than 65 years. However, it was effective in other subgroups. Detailed results are provided in Table 2.

**TABLE 2:** The subgroup results.

Subgroup	Overall efficacy				Change in NIHSSS			
	Study	RR [95% CI]	*P* Value	*I* ^2^	Study	SMD [95% CI]	*P* Value	*I* ^2^
Total	35	1.20 [1.16–1.25]	<0.00001	49%	21	−0.42 [−0.77 to −0.07]	0.02	95%
Patients								
Simple acute stroke	21	1.22 [1.18–1.27]	<0.00001	22%	15	−0.62 [−0.87 to −0.37]	<0.00001	88%
Stroke with diabetes	2	1.15 [1.02–1.29]	0.02	0%	2	−2.14 [−3.13 to −1.15]	<0.0001	87%
Intervention time								
>4 weeks	13	1.18 [1.12–1.25]	<0.00001	48%	6	−0.44 [−1.08 to 0.19]	0.17	96%
≤4 weeks	19	1.21 [1.15–1.28]	<0.00001	57%	12	−0.72 [−1.10 to −0.33]	0.0003	93%
Mean/median age								
>65 years	4	1.13 [1.06–1.20]	0.0002	0%	4	0.68 [−1.47 to 2.83]	0.53	99%
≤65 years	25	1.20 [1.15–1.26]	<0.00001	55%	16	−0.68 [−0.96 to −0.40]	<0.00001	91%

## 4 Discussion

Tongxinluo capsules are a traditional and safe Chinese medicine used effectively for the secondary prevention of coronary artery diseases. Approved by the China Food and Drug Administration over 20 years ago, it is indicated for the treatment of coronary heart disease, angina, ischemic stroke, and related conditions (Zhuo et al., 2008; Wang and Liu, 2024). A 2015 study by Junlei Di, which included 144 patients, found that Tongxinluo showed significant efficacy in treating acute cerebral infarction (Di, 2015). Tongxinluo is commonly used as an adjuvant in stroke treatment, co-administered with agents such as aspirin, clopidogrel, edaravone, butylphthalide, and statins to achieve synergistic therapeutic effects.

Our study found that Tongxinluo significantly improved the overall therapeutic effect, NIHSS score, TC, TG, hs-CRP, LDL, HDL, TNF-α, IL-6, high-shear and low-shear whole blood viscosity, Barthel index, plaque area, MMSE score, FM score, plasma viscosity, plasma fibrinogen, hematocrit, NO, and quality of life index. However, no significant effects were observed in TG, IL-18, and platelet aggregation function. Sensitivity analysis revealed instability in FM score, MMSE score, NIHSS score, TG, and TNF-α. Egger’s test for overall efficacy, LDL, HDL, TNF-α, and MMSE score indicated publication bias. A 2024 study by Yi Dong (Dong et al., 2024), which included 1,946 patients, demonstrated that after 90 days of treatment, Tongxinluo significantly treated acute ischemic stroke. Our findings align with Yi Dong’s results and further validate the efficacy and safety of Tongxinluo in adjunctive stroke treatment, providing evidence to support its clinical application.

Subgroup analysis revealed that the effect was not significant when the intervention duration exceeded 4 weeks, likely due to patient adherence issues. Long-term use of Tongxinluo may cause adverse reactions, including gastrointestinal discomfort, nausea, vomiting, bloating, and diarrhea, potentially resulting from gastrointestinal irritation. Allergic reactions such as rashes and itching, as well as headaches and dizziness, may be linked to its impact on the vascular and nervous systems (Li P. et al., 2021). Prolonged use can decrease adherence, leading to irregular medication usage and affecting treatment outcomes (Lv et al., 2022). Therefore, based on this study’s findings, it is recommended that Tongxinluo be used for no more than 4 weeks in clinical adjunctive stroke treatment. For patients over 65, Tongxinluo showed no significant effect on NIHSS scores, likely due to the presence of multiple underlying health conditions that may reduce its efficacy (Dong et al., 2024).

Tongxinluo enhances myocardial reperfusion, reduces no-reflow incidence, and decreases infarct size, thereby improving heart function. It also exhibits anti-atherosclerotic effects by stabilizing plaques and preventing their progression. Its mechanisms of action include regulating cytophysical functions, hormone secretion, protein binding, immune responses, inflammation, and improving lipid metabolism (Liu et al., 2023). The diverse therapeutic effects of Tongxinluo contribute to its potential in stroke treatment and support its clinical application in cerebrovascular disease management. Studies indicate that Tongxinluo capsules effectively regulate blood lipids, prevent coagulation, and stabilize plaques. Modern pharmacological research reveals that components such as scorpion and leech possess anticoagulant effects, inhibiting thrombosis and preventing atherosclerosis, while also improving coronary blood flow. Additionally, cicada slough effectively suppresses platelet aggregation and regulates blood rheology, preventing thrombosis. This drug not only enhances myocardial contractility and restores heart function but also inhibits platelet aggregation, regulates blood lipid levels, and improves prognosis.

The main active ingredients in Tongxinluo, including flavonoids and saponins, reduce platelet activation and aggregation, lowering thrombosis risk. By inhibiting thrombin activity and regulating the fibrinolytic system, it mitigates the blood’s hypercoagulable state. Tongxinluo also reduces vascular endothelial inflammation by inhibiting inflammatory factors such as TNF-α, IL-1β, and IL-6. Components like ginsenosides and ligustrazine scavenge free radicals and reduce oxidative stress. Furthermore, Tongxinluo enhances endothelial cell proliferation and migration, aiding in endothelial repair. By inhibiting oxidative stress and mitochondrial apoptosis pathways, it protects endothelial cells and reduces apoptosis. It also dilates coronary arteries, improving myocardial blood flow, alleviating myocardial ischemia and hypoxia, and inhibiting myocardial remodeling (Wei and Jiang, 2023). Tongxinluo capsules have been reported to modulate lipid metabolism, exhibit anticoagulant activity, and stabilize atherosclerotic plaques. Pharmacological studies suggest that principal components such as Chinese scorpion (Girardinia diversifolia subsp. diversifolia) and medicinal leech (Cucumis melo L.) possess potent anticoagulant and antithrombotic effects, attenuate atherosclerotic progression, and enhance coronary perfusion. Cicada Molt (Salvia miltiorrhiza Bunge) has likewise been shown to inhibit platelet aggregation, improve hemorheological parameters, and mitigate thrombosis risk. Frankincense (*Boswellia sacra Flück.*), rich in boswellic acids, suppresses proinflammatory cytokines such as TNF-α and IL-6, thereby alleviating neuroinflammation. It also reduces blood viscosity, inhibits platelet aggregation, and prevents thrombosis. Ginseng (Panax ginseng C.A.Mey.*)* provides ginsenosides (e.g., Rg1, Rb1), which promote neural stem cell proliferation and differentiation, facilitate neural pathway repair, enhance endothelial function, suppress vascular smooth muscle proliferation, and stabilize atherosclerotic plaques. White peony root (Paeonia lactiflora Pall). Contains Albiflorin, which inhibits platelet adhesion and aggregation, lowers fibrinogen levels, and suppresses the NF-κB pathway, reducing infiltration of inflammatory cells such as neutrophils and macrophages in the brain. Sour jujube seed (Ziziphus jujuba Mill). Contains jujubosides and flavonoids that regulate the GABAergic system, relieve post-stroke anxiety and insomnia, inhibit glutamate-induced excitotoxicity, and prevent neuronal overactivation. Volatile oils in sandalwood (Santalum album L.*)* facilitate the flow of qi, alleviate blood stasis, dilate cerebral vessels, and enhance perfusion, particularly in ischemic regions. Borneo [Blumea balsamifera (L.) DC]. Contains small lipophilic molecules that increase blood–brain barrier permeability, improving the delivery of active compounds to the brain and enhancing synergistic effects. Chinese cockroach (*E. sinensis Walker)* provides urokinase-like components that promote thrombolysis and upregulate vascular endothelial growth factor (VEGF), facilitating collateral circulation and improving cerebral perfusion. Centipede (*S. subspinipes mutilans* L. Koch) modulates TRPV1 ion channels to relieve neuropathic pain, enhance nerve conduction, and improve motor dysfunction. Moreover, Tongxinluo may strengthen myocardial contractility, restore cardiac pump function, regulate lipid profiles, and contribute to improved clinical outcomes.

The principal bioactive components of Tongxinluo, including flavonoids and saponins, inhibit platelet activation and aggregation, thereby lowering the risk of thrombosis. By suppressing thrombin activity and modulating the fibrinolytic system, Tongxinluo ameliorates the hypercoagulable state. It further reduces vascular inflammation by downregulating pro-inflammatory cytokines such as TNF-α, IL-1β, and IL-6. Ginsenosides and ligustrazine demonstrate potent antioxidant properties by scavenging free radicals and attenuating oxidative stress. Tongxinluo enhances endothelial cell proliferation and migration, facilitates vascular repair, and maintains endothelial integrity by inhibiting oxidative stress and mitochondrial apoptotic pathways. Moreover, it promotes coronary vasodilation and improves myocardial perfusion, thereby alleviating ischemia and hypoxia and mitigating adverse cardiac remodeling (Liu et al., 2008a).

Tongxinluo capsules contain key active ingredients such as ginseng (Panax ginseng C.A.Mey.*)* (vasodilatory), medicinal leech (Cucumis melo L.) (leech extract, antiplatelet), and Borneo [Blumea balsamifera (L.) DC]. (borneol, circulation-enhancing and analgesic). The formulation is grounded in meridian pathology theory to enhance its ability to open channels, activate blood flow, reduce vascular resistance, and improve systemic perfusion. Tongxinluo provides neuroprotection by inhibiting mitochondrial apoptotic enzymes, thereby restoring cellular metabolism, enhancing regional blood flow, and preserving cerebral function. Clinical studies have demonstrated that Tongxinluo dilates cerebral vessels, reduces blood viscosity as well as levels of total cholesterol and triglycerides, prevents cerebral perfusion injury, and supports cerebral metabolism and neurological recovery (Liu et al., 2008b).

This study has several limitations. First, significant heterogeneity exists due to considerable variation in sample sizes across the included studies. Secondly, the majority of included studies provided insufficient detail regarding blinding and allocation methods, as indicated by the quality assessment charts. These methodological shortcomings may have introduced selection and information bias, thereby complicating the evaluation of Tongxinluo’s efficacy and safety. In response, we employed more rigorous analytical approaches and critically assessed each phase of the research process. Through comprehensive and multidimensional analysis, we achieved a more structured and in-depth understanding of Tongxinluo’s therapeutic role in stroke management. To mitigate the impact of inadequate reporting, we implemented several methodological strategies. Stratified and sensitivity analyses were conducted to examine the data from multiple perspectives and minimize potential bias. During the literature search, we prioritized the acquisition of more detailed study information. These measures will inform future methodological improvements and contribute to the production of more reliable and impactful research outcomes. Many studies retrieved from Chinese databases are single-center with limited sample sizes, which may introduce publication and regional selection biases. Additional large-scale, high-quality studies are required to enhance the strength of current evidence. The lack of detailed data on stroke localization in the original studies precluded subgroup analyses based on specific vascular territories. Consequently, whether Tongxinluo exerts consistent effects across different infarct locations remains uncertain and warrants further investigation. Moreover, several studies provided insufficient information regarding placebo use and its potential impact, limiting the accuracy of efficacy assessments. Placebo responses may bias symptom reporting, potentially masking or amplifying actual treatment effects. Future trials should specify placebo protocols in detail—including composition, appearance, and administration—to align with the Tongxinluo group, and employ double- or triple-blind designs to minimize subjective bias. Population characteristics and age were identified as primary contributors to heterogeneity. However, limited data availability hindered subgroup analyses of other potential effect modifiers. Based on clinical insights and prior evidence, factors such as stroke type, sex, and sample size may also influence heterogeneity, though further validation is required. The included interventions were highly variable, with routine treatments involving complex combinations of traditional Chinese and Western medicines. Due to this diversity, systematic classification was not feasible. Future studies should adopt more targeted designs to clarify these effects, control for confounding variables, and enhance the overall quality of evidence.

## 5 Conclusion

Our study demonstrated that Tongxinluo, as an adjunctive therapy, significantly improved overall efficacy, NIHSS score, TC, hs-CRP, LDL, HDL, TNF-α, IL-6, high-shear whole blood viscosity, low-shear whole blood viscosity, Barthel index score, plaque area, MMSE score, FM score, plasma viscosity, plasma fibrinogen, red blood cell volume, NO, and quality of life index in stroke patients. Tongxinluo appears to be an effective and safe adjunctive treatment for stroke. Subgroup analysis showed better efficacy in patients aged ≤65 years and with an intervention duration of ≤4 weeks. Given the predominance of studies from China and the lack of data from other countries, as well as potential heterogeneity and publication bias, further international, multicenter RCTs are required to confirm Tongxinluo’s efficacy, safety, and potential influencing factors in stroke treatment.

## Data Availability

The original contributions presented in the study are included in the article/Supplementary Material, further inquiries can be directed to the corresponding author.
