# Supplementary material for: Efficacy and safety of Tongxinluo in the treatment of stroke: a systematic review and meta-analysis of randomized controlled trials
Source: Front Pharmacol. 2025 Jul 21;16:1573069. doi: 10.3389/fphar.2025.1573069 (PMC12318962; doi:10.3389/fphar.2025.1573069)
Supplement: Supplementary file 1 [file Supplementaryfile1.docx]

**Supplementary materials**

**Table S1** Searching strategy details

| Databases | Searching strategy | Number of literature |
| --- | --- | --- |
| Pubmed | ((("tongxinluo" [Supplementary Concept]) OR (Tongxinluo)) AND (("Stroke, Lacunar"[Mesh]) OR (((((((((((((((((Strokes) OR (Cerebrovascular Accident)) OR (Cerebrovascular Accidents)) OR (Cerebral Stroke)) OR (Cerebral Strokes)) OR (Cerebrovascular Apoplexy)) OR (Brain Vascular Accident)) OR (Brain Vascular Accidents)) OR (Cerebrovascular Stroke)) OR (Cerebrovascular Stroke)) OR (Apoplexy)) OR (CVA)) OR (CVAs)) OR (Acute Stroke)) OR (Acute Strokes)) OR (Acute Cerebrovascular Accident)) OR (Acute Cerebrovascular Accidents)))) AND (random*) | 27 |
| Embase | (tongxinluo or Tongxinluo) and (Stroke, Lacunar or (Strokes or Cerebrovascular Accident or Cerebrovascular Accidents or Cerebral Stroke or Cerebral Srokes orCerebrovascular Apoplexy or Brain Vascular Accident or Brain VascuarAccidenls or Cerebrovascuar Stroke or Cerebrovascular Stroke orApoplexy or CVA or CVAs or AcuteStroke or Acute Strokes or Acute Cerebrovascular Accident or Acute Cerebrovascular Accidents)) and random*).af. | 16 |
| Cochrane | (tongxinluo or Tonxinluo) and (Sroke, Lacunar or (Stokes or Cerebrovascular Accident or Cerebrovascuar Accidenis or cerebral Stoke or Cerebral Strokes orCerebrovascular Apoplexy or Brain ascular Accident or Brain yascularAccidents or Cerebrovascular Stroke or Cerebrovascular Stoke or Apoplexy or CVA o CVAs or AcuteStroke or Acute Strokes or Acute Cerebrovascular Accident or Acute Cerebrovascular Accidents)) and random*).af. | 6 |
| Web of science | (((tongxinluo) OR (Tongxinluo)) AND ((Stroke, Lacunar) OR (((((((((((((((((Strokes) OR (Cerebrovascular Accident)) OR (Cerebrovascular Accidents)) OR (Cerebral Stroke)) OR (Cerebral Strokes)) OR (Cerebrovascular Apoplexy)) OR (Brain Vascular Accident)) OR (Brain Vascular Accidents)) OR (Cerebrovascular Stroke)) OR (Cerebrovascular Stroke)) OR (Apoplexy)) OR (CVA)) OR (CVAs)) OR (Acute Stroke)) OR (Acute Strokes)) OR (Acute Cerebrovascular Accident)) OR (Acute Cerebrovascular Accidents)))) AND (random*) | 20 |
| Wanfang | 通心络AND （脑卒中 OR 卒中 OR 脑梗死 OR 脑埂塞） AND 随机 | 156 |
| CNKI | （通心络[篇关摘]）AND （脑卒中[篇关摘] OR 卒中[篇关摘] OR 脑梗死[篇关摘] OR 脑埂塞[篇关摘]） AND （随机[篇关摘]） | 267 |
| VIP | （通心络[摘要]）AND （脑卒中[摘要] OR 卒中[摘要] OR 脑梗死[摘要] OR 脑埂塞[摘要]） AND （随机[摘要]） | 309 |
| CBM | “通心络"[摘要:智能] AND("卒中"[摘要:智能]OR"脑梗死"[摘要:智能]  OR"脑梗寒"[摘要:智能]) AND"随机"[摘要:智能] | 293 |

**Table S2** The main components of Tongxinluo.

| Ingredients of Tongxinluo Capsules | Pinyin | Latin Name | Functions | Adverse reaction | Pharmacokinetics |
| --- | --- | --- | --- | --- | --- |
| 乳香 | Rǔ Xiānɡ | [*Boswellia sacra Flück.*](https://mpns.science.kew.org/mpns-portal/plantDetail?plantId=2680579&query=%E4%B9%B3%E9%A6%99&filter=&fuzzy=false&nameType=all&dbs=wcsCmp) | Invigorates blood, alleviates pain, reduces swelling, promotes tissue regeneration. | Gastrointestinal discomfort: Oral intake of frankincense may irritate the gastrointestinal tract, causing symptoms such as nausea, vomiting, abdominal pain, and diarrhea. | The pharmacokinetics of Boswellia carteri Birdw are primarily associated with the absorption, distribution, metabolism, and excretion of its active constituents, such as boswellic acids, volatile oils, and pentacyclic triterpenoids. Following oral administration, the bioavailability of boswellic acids—particularly acetyl-11-keto-β-boswellic acid (AKBA)—is relatively low (approximately 1%), likely due to first-pass metabolism and poor water solubility. Absorption occurs mainly via passive diffusion in the intestine, with a high plasma protein binding rate (>90%). Distribution predominantly targets the liver and inflamed tissues. Metabolism occurs mainly in the liver through cytochrome P450 enzymes (e.g., CYP3A4)-mediated oxidation and uridine 5'-diphospho-glucuronosyltransferase (UGT)-mediated glucuronidation, producing hydroxylated and conjugated metabolites. Excretion is primarily fecal (approximately 70%), with a small proportion eliminated via urine. The elimination half-life is estimated to be around 6–8 hours. |
| 降香 | Jiànɡ Xiānɡ | [*Dalbergia odorifera T.C.Chen*](https://mpns.science.kew.org/mpns-portal/plantDetail?plantId=2755780&query=%E9%99%8D%E9%A6%99&filter=&fuzzy=false&nameType=all&dbs=wcs) | Resolvess blood stasis, stops bleeding, regulates Qi, relieves pain. | Gastrointestinal reactions: Oral administration of Dalbergia odorifera (Jiangxiang) may irritate the gastrointestinal tract. A small number of individuals may experience symptoms such as nausea, vomiting, stomach discomfort, or mild diarrhea.  Allergic reactions: In rare cases, individuals with allergic constitutions may develop hypersensitivity reactions after contact with or ingestion of Dalbergia odorifera, including skin itching, rashes, or urticaria. In severe instances, symptoms such as difficulty breathing may occur, although such reactions are uncommon. | The pharmacokinetics of Dalbergia odorifera are primarily associated with its active constituents, including flavonoids, volatile oils, and sesquiterpenes. Following oral administration, flavonoids such as naringenin and luteolin, as well as volatile components like trans-nerolidol, are rapidly absorbed in the small intestine, exhibiting moderate bioavailability (approximately 20%–40%). Their absorption is influenced by first-pass hepatic metabolism and intestinal microbiota activity. After absorption, these compounds are widely distributed across the liver, lungs, and heart, with a high plasma protein binding rate (>80%).  Metabolism occurs mainly in the liver via cytochrome P450 enzymes, particularly CYP3A4 and CYP2C9, through hydroxylation and methylation reactions. Some flavonoid glycosides undergo hydrolysis by intestinal microbiota into aglycones, which are subsequently absorbed. The primary routes of excretion are through urine (approximately 60%) and feces (approximately 30%). The elimination half-life is estimated to be 4–6 hours.  The cardiovascular protective effects of Dalbergia odorifera are closely linked to the antioxidant and anti-inflammatory properties of its active compounds and their metabolites. |
| 人参 | Rén Shēn | [*Panax ginseng C.A.Mey.*](https://mpns.science.kew.org/mpns-portal/plantDetail?plantId=146697&query=%E4%BA%BA%E5%8F%82&filter=&fuzzy=false&nameType=all&dbs=wcs) | Greatly tonifies original Qi, restores pulses, strengthens the spleen and lungs, generates fluids, nourishes blood, calms the mind, and improves cognition. | Central nervous system stimulation: Excessive intake may lead to central nervous system excitation, resulting in symptoms such as insomnia, vivid dreams, headache, dizziness, palpitations, and restlessness. Some individuals may also experience elevated blood pressure.  Gastrointestinal discomfort: Digestive symptoms such as nausea, vomiting, diarrhea, and loss of appetite may occur. | The pharmacokinetics of Panax ginseng are primarily associated with its active components—ginsenosides such as Rb1, Rg1, and Re—regarding their absorption, distribution, metabolism, and excretion. Following oral administration, the bioavailability of ginsenosides is generally low (e.g., Rb1 approximately 1–5%, Rg1 approximately 10–20%), largely due to first-pass metabolism and extensive biotransformation by intestinal microbiota, including hydrolysis into aglycones such as compound K.  After absorption, ginsenosides are widely distributed in the liver, kidneys, heart, and brain. Notably, Rb1 exhibits greater lipophilicity, facilitating its penetration into brain tissue. Metabolism primarily occurs via hepatic cytochrome P450 enzymes (e.g., CYP3A4) and gut microbiota-mediated hydrolysis, producing secondary ginsenosides (e.g., Rd, F1) and aglycones (e.g., protopanaxadiol and protopanaxatriol).  Excretion is mainly through urine (30–50%) and feces (40–60%). The elimination half-life varies significantly depending on the ginsenoside type—approximately 16–20 hours for Rb1 and 5–8 hours for Rg1. |
| 白芍 | Bái Sháo | [*Paeonia lactiflora Pall.*](https://mpns.science.kew.org/mpns-portal/plantDetail?plantId=519125&query=%E7%99%BD%E8%8A%8D&filter=&fuzzy=false&nameType=all&dbs=wcs) | Nourishes blood, regulates menstruation, restrains Yin, stops sweating, soothes the liver, relieves pain, and pacifies liver Yang. | Effects on coagulation function: Due to its properties of promoting blood circulation and removing blood stasis, it may inhibit platelet aggregation and prolong coagulation time, thereby increasing the risk of bleeding. Caution is advised in individuals with coagulation disorders or those undergoing surgery.  Effects on the central nervous system: The pharmacological effects may mildly suppress the central nervous system, leading to symptoms such as dizziness, drowsiness, and general fatigue in some individuals. Caution should be exercised when driving or operating machinery. | The pharmacokinetics of Paeonia lactiflora are primarily associated with its active constituents, particularly paeoniflorin and other monoterpene glycosides. After oral administration, paeoniflorin is rapidly absorbed but exhibits low bioavailability (approximately 3%–10%), mainly due to intestinal microbial metabolism (e.g., hydrolysis into metabolite-I) and P-glycoprotein-mediated efflux.  Following absorption, paeoniflorin is widely distributed in the liver, kidneys, and spleen, though it has limited ability to cross the blood–brain barrier. Its plasma protein binding rate ranges from approximately 30% to 50%. Metabolism primarily involves hydrolysis by intestinal microbiota and glucuronidation by hepatic UGT enzymes, producing deglycosylated or conjugated metabolites.  Excretion occurs predominantly via the urine (approximately 60%–70%), while a portion of the parent compound and its metabolites is excreted via bile into the feces. The elimination half-life is approximately 1.5–3 hours. |
| 酸枣仁 | Suān Zǎo Rén | [*Ziziphus jujuba Mill.*](https://mpns.science.kew.org/mpns-portal/plantDetail?plantId=2470699&query=%E9%85%B8%E6%9E%A3%E4%BB%81&filter=&fuzzy=false&nameType=all&dbs=wcsCmp) | Nourishes the heart, tonifies the liver, calms the mind, and spirit, restrains sweating, generates fluids. | Gastrointestinal discomfort: Excessive consumption or use by individuals with gastrointestinal weakness may affect gastric acid secretion and gastrointestinal motility, leading to symptoms such as stomach pain, nausea, vomiting, and diarrhea. Due to the moist and lubricating nature of Ziziphus jujuba seed (Suanzaoren), large doses of the single herb may worsen symptoms in those prone to loose stools.  Abnormal blood pressure: It may suppress cardiac function and lower blood pressure. In individuals with cardiac conduction block or hypotension, high doses may worsen the condition, leading to symptoms such as dizziness and fatigue related to low blood pressure. | The pharmacokinetics of Ziziphus jujuba var. spinosa are primarily related to its active components, including saponins (e.g., jujuboside A and B), flavonoids (e.g., spinosin), and alkaloids. After oral administration, flavonoids such as spinosin are absorbed relatively quickly, with moderate bioavailability (approximately 15%–30%). In contrast, saponins exhibit poor absorption (<5%) due to low permeability and hydrolysis by intestinal microbiota.  Both parent compounds and their metabolites are widely distributed in organs such as the heart, liver, and brain. Notably, spinosin is capable of crossing the blood–brain barrier. Metabolism occurs primarily in the liver via cytochrome P450 (CYP) enzyme-mediated oxidation and uridine 5′-diphospho-glucuronosyltransferase (UGT)-mediated conjugation. A key metabolic pathway involves intestinal microbial hydrolysis, such as the conversion of jujuboside A into its aglycone jujubogenin.  Excretion occurs mainly through urine (40%–60%) and feces (30%–50%). The elimination half-life of spinosin is approximately 4–6 hours, while that of saponins is longer (8–12 hours) due to differences in metabolic rates. |
| 檀香 | Tán Xiānɡ | [*Santalum album L.*](https://mpns.science.kew.org/mpns-portal/plantDetail?plantId=2581919&query=%E6%AA%80%E9%A6%99&filter=&fuzzy=false&nameType=all&dbs=wcsCmp) | Moves Qi, warms the middle, opens the appetite, and relieves pain. | Digestive system discomfort: Oral administration of sandalwood (Santalum album) may cause digestive reactions in some individuals, such as nausea, vomiting, epigastric discomfort, or acid regurgitation, potentially affecting normal spleen and stomach function.  Allergic reactions: Individuals with allergic constitutions may develop skin-related allergic symptoms—such as itching, rashes, or urticaria—after contact with or use of products containing sandalwood. | The pharmacokinetics of Santalum album are primarily associated with its volatile constituents, including α-santalol, β-santalol, and other sesquiterpenoids. Following oral administration, santalol compounds are rapidly absorbed, exhibiting moderate bioavailability (approximately 20%–40%). However, their absorption is significantly affected by first-pass hepatic metabolism, primarily through CYP2C9 and CYP3A4, resulting in hydroxylated metabolites.  Due to their high lipophilicity, these compounds are widely distributed in adipose tissue, liver, and the central nervous system, and are capable of crossing the blood–brain barrier. Metabolites are mainly excreted via urine (approximately 50%–70%) and exhalation, with some parent compounds also eliminated through the lungs. The elimination half-life is approximately 3–6 hours. |
| 冰片 | Bīnɡ Piàn | [*Blumea balsamifera (L.) DC.*](https://mpns.science.kew.org/mpns-portal/plantDetail?plantId=2906014&query=%E5%86%B0%E7%89%87&filter=&fuzzy=false&nameType=all&dbs=wcsCmp) | Opens the orifices, clears the mind, clears heat, and alleviates pain. | Systemic reactions: In rare cases, oral administration of borneol (e.g., in traditional Chinese patent medicines) may cause laryngeal edema, dyspnea, chest tightness, or even anaphylactic shock, requiring immediate medical intervention.  Adverse oral reactions: Due to its bitter and cold nature, excessive oral intake of borneol may irritate the gastrointestinal mucosa, leading to nausea, vomiting, abdominal pain, diarrhea, and in severe cases, gastrointestinal bleeding. | The pharmacokinetics of synthetic borneol are primarily related to its active monoterpenoid constituents, such as borneol and isoborneol. After oral administration, absorption is rapid and nearly complete, with relatively high bioavailability (approximately 60%–80%). However, it is subject to first-pass hepatic metabolism, primarily via cytochrome P450 enzymes such as CYP2C19, producing hydroxylated metabolites.  Due to its high lipophilicity, synthetic borneol readily crosses the blood–brain barrier and is widely distributed in the brain, heart, liver, and other tissues. The plasma protein binding rate is approximately 70%–90%. Metabolites are mainly excreted in the urine as glucuronide conjugates (approximately 80%–90%), with small amounts eliminated via respiration or perspiration. The elimination half-life is approximately 1–3 hours. |
| 水蛭 | Shuǐ Zhì | [*Cucumis melo L.*](https://mpns.science.kew.org/mpns-portal/plantDetail?plantId=2746992&query=%E6%B0%B4%E8%9B%AD&filter=&fuzzy=true&nameType=all&dbs=wcsCmp) | Breaks blood stasis, unblocks the meridians, expels blood stasis, and resolves masses. | minal pain, and diarrhea. Prolonged or high-dose use may lead to gastrointestinal bleeding.  Liver and kidney function impairment: Long-term or excessive use may affect liver and kidney function, potentially resulting in abnormal liver function indicators (e.g., elevated transaminases) and changes in urine output. | The pharmacokinetics of Hirudo nipponica are chiefly determined by its active constituents—hirudin and hirudin-like polypeptides. When taken orally, these large peptides undergo extensive gastrointestinal degradation, resulting in extremely low bioavailability (<1%); consequently, parenteral administration is required, yielding nearly 100 % bioavailability. In the bloodstream, hirudin exerts direct anticoagulant activity. It displays a small apparent volume of distribution, remaining largely within the plasma where it forms stable complexes with thrombin.  Elimination occurs predominantly via renal clearance, with the unchanged molecule accounting for roughly 80 %–90 % of urinary excretion. The elimination half-life is approximately 1–2 hours following intravenous injection. Certain smaller active peptides (e.g., desirudin) can be partially absorbed after oral administration, but their systemic availability is markedly reduced by extensive first-pass metabolism. |
| 蝉蜕 | Chán Tuì | [*Salvia miltiorrhiza Bunge*](https://mpns.science.kew.org/mpns-portal/plantDetail?plantId=183206&query=%E8%9D%89%E8%9C%95&filter=&fuzzy=true&nameType=all&dbs=wcs) | Disperses wind-heat, benefits the throat, expels rashes, clears vision and removes eye obstruction, stops wind and relieves spasms. | Effects on the nervous system: Cicada molting (Chantui) has a mild sedative effect. Excessive intake may suppress the central nervous system, leading to dizziness, drowsiness, and fatigue. Caution should be taken when driving or operating machinery to avoid accidents due to slowed reactions.  Allergic reactions: Individuals with allergic constitutions may be sensitive to proteins or other components in Cicada molting, resulting in symptoms such as skin itching, rashes, or urticaria. In severe cases, laryngeal edema, breathing difficulty, or even anaphylactic shock may occur. | The pharmacokinetics of Cryptotympana pustulata are primarily associated with its active components, including chitin, proteins, and trace elements. Following oral administration, large molecular substances such as chitin exhibit extremely low bioavailability due to poor gastrointestinal absorption. These macromolecules must first be degraded by intestinal microbiota into low-molecular-weight products (e.g., chitooligosaccharides), which can then be partially absorbed.  Small-molecule bioactive compounds, such as amino acids and trace elements, are absorbed through the intestinal tract with a bioavailability of approximately 10%–30%. After absorption, the components are widely distributed in the liver, kidneys, and immune-related tissues. Chitin-derived metabolites may interact with immune cells, such as macrophages.  Metabolism primarily involves microbial degradation in the gut and hepatic processing. Excretion occurs mainly via feces (approximately 70%–80%), with a small proportion eliminated in the urine. |
| 全蝎 | Quán Xiē | [*Girardinia diversifolia subsp. diversifolia*](https://mpns.science.kew.org/mpns-portal/plantDetail?plantId=3264768&query=%E5%85%A8%E8%9D%8E&filter=&fuzzy=true&nameType=all&dbs=wcsCmp) | Subdues wind, relieves spasms, unblocks meridians, relieves pain, clears toxins, and disperses masses. | Skin and mucosal symptoms: Individuals with allergic constitutions may be allergic to scorpion protein, resulting in symptoms such as rashes, itching, and urticaria. In severe cases, laryngeal edema and breathing difficulties may occur.  Liver and kidney function impairment: Scorpion toxins are metabolized by the liver and kidneys. Long-term or high-dose use may lead to liver and kidney dysfunction, manifested by elevated transaminases, increased serum creatinine, and even acute hepatic or renal failure. Individuals with preexisting liver or kidney insufficiency are at higher risk of toxin accumulation and poisoning. | The pharmacokinetics of Buthus martensii are primarily associated with its active components, including scorpion venom peptides (such as antiepileptic and analgesic peptides) and protein-based substances. Following oral administration, large polypeptides are easily degraded by the gastrointestinal tract, resulting in extremely low bioavailability (<5%). Therefore, parenteral administration (with bioavailability >90%) or advanced delivery systems (e.g., enteric-coated capsules) are typically required to enhance absorption.  Small-molecule components, such as amino acids and organic acids, can be absorbed through the intestinal tract with a bioavailability of approximately 15%–25%. After absorption, the compounds are widely distributed in the liver, kidneys, and nervous system. Some bioactive peptides are capable of crossing the blood–brain barrier.  Metabolism primarily involves proteolytic degradation and renal clearance. Both parent compounds and metabolites are mainly excreted via the urine (approximately 60%–80%). The elimination half-life is approximately 2–4 hours following intravenous administration. |
| 土鳖虫 | Tǔ Biē Chóng | *Eupolyphaga sinensis* Walker | Breaks blood stasis, expels stasis, restores tendons, and connects bones. | Increased risk of bleeding: *Eupolyphaga* (Tuban Chong) is known for promoting blood circulation and removing blood stasis. Excessive or prolonged use may inhibit platelet aggregation, leading to subcutaneous bleeding (ecchymosis, purpura), gum bleeding, and nosebleeds. In women, it may cause increased menstrual flow, prolonged menstruation, or even metrorrhagia. In individuals with gastric ulcers or coagulation disorders, it may trigger gastrointestinal bleeding or delay wound healing. | The pharmacokinetics of Steleophaga plancyi are chiefly attributed to its active constituents—amino acids, peptides, unsaturated fatty acids, and trace elements. Following oral administration, large polypeptides undergo extensive gastrointestinal degradation, resulting in low bioavailability (<10%). In contrast, small-molecule substances such as amino acids and nucleosides are absorbed more rapidly, with bioavailability of roughly 20 %–40 %.  After absorption, these components are distributed mainly to the liver, kidneys, and bone tissue. Certain constituents can traverse the blood–bone barrier, thereby participating in the regulation of bone metabolism. Metabolic processing involves hepatic enzymatic degradation and transformations mediated by the intestinal microbiota. Elimination occurs primarily via urine (50 %–70 %) and feces (30 %–50 %). The elimination half-life is approximately 3–6 hours. |
| 蜈蚣 | Wú Gōnɡ | *Scolopendra subspinipes mutilans* L. Koch | Subdues wind, relieves spasms, unblocks meridians, relieves pain, clears toxins, and disperses masses. | Neurotoxicity: *Centipede* toxins can act directly on the central and peripheral nervous systems. Overdose may cause dizziness, headache, numbness of the mouth and tongue, and limb convulsions (e.g., facial muscle twitching, limb rigidity). In severe cases, symptoms may progress to ataxia, tonic spasms, coma, and even death due to respiratory center paralysis.  Respiratory system: Individuals with allergic constitutions may experience laryngeal edema, dyspnea, chest tightness, and shortness of breath. In severe cases, anaphylactic shock may occur. | The pharmacokinetics of Scolopendra subspinipes mutilans are primarily associated with its active components, including toxic proteins, peptides, histamine, and hemolytic substances. Following oral administration, large protein and polypeptide constituents are readily degraded in the gastrointestinal tract, resulting in extremely low bioavailability (<5%). Processing techniques to reduce toxicity or parenteral administration are typically required to improve systemic availability. In contrast, small-molecule compounds such as amino acids and nucleosides can be partially absorbed, with bioavailability ranging from approximately 15%–30%.  After absorption, these components are primarily distributed in the liver, kidneys, and joint tissues. Some active compounds may preferentially accumulate at sites of inflammation. Metabolism mainly involves hepatic degradation, while excretion occurs predominantly via renal pathways. Both parent compounds and metabolites are eliminated mainly through urine (60%–80%). The elimination half-life of small-molecule constituents is approximately 2–5 hours. |
| Adverse Reactions of Tongxinluo | Gastrointestinal discomfort: This is relatively common and may include symptoms such as stomach discomfort, nausea, vomiting, abdominal pain, diarrhea, and loss of appetite, mostly due to irritation of the gastrointestinal mucosa by the drug components. Individuals with weak spleen and stomach function are more susceptible. Taking the medication after meals may help reduce these symptoms to some extent.  Allergic reactions: A small number of patients may develop rashes, skin itching, or redness. In severe cases, symptoms such as difficulty breathing or tachycardia may occur. Caution is advised in individuals with allergic constitutions, and those known to be allergic to the drug's components should avoid its use.  Other potential effects: Some individuals may experience dizziness or headache, possibly due to effects on the nervous system or cerebral vascular regulation. Cardiovascular abnormalities such as palpitations or blood pressure fluctuations may occur. There may also be a tendency for bleeding (e.g., gum bleeding, nosebleeds, subcutaneous bruising), likely related to the drug's blood-activating properties. Long-term or high-dose use may increase the burden on the liver and kidneys, potentially affecting their function and leading to symptoms such as liver area pain or lower limb edema. However, these effects are relatively rare and may vary between individuals. | | | | |

| 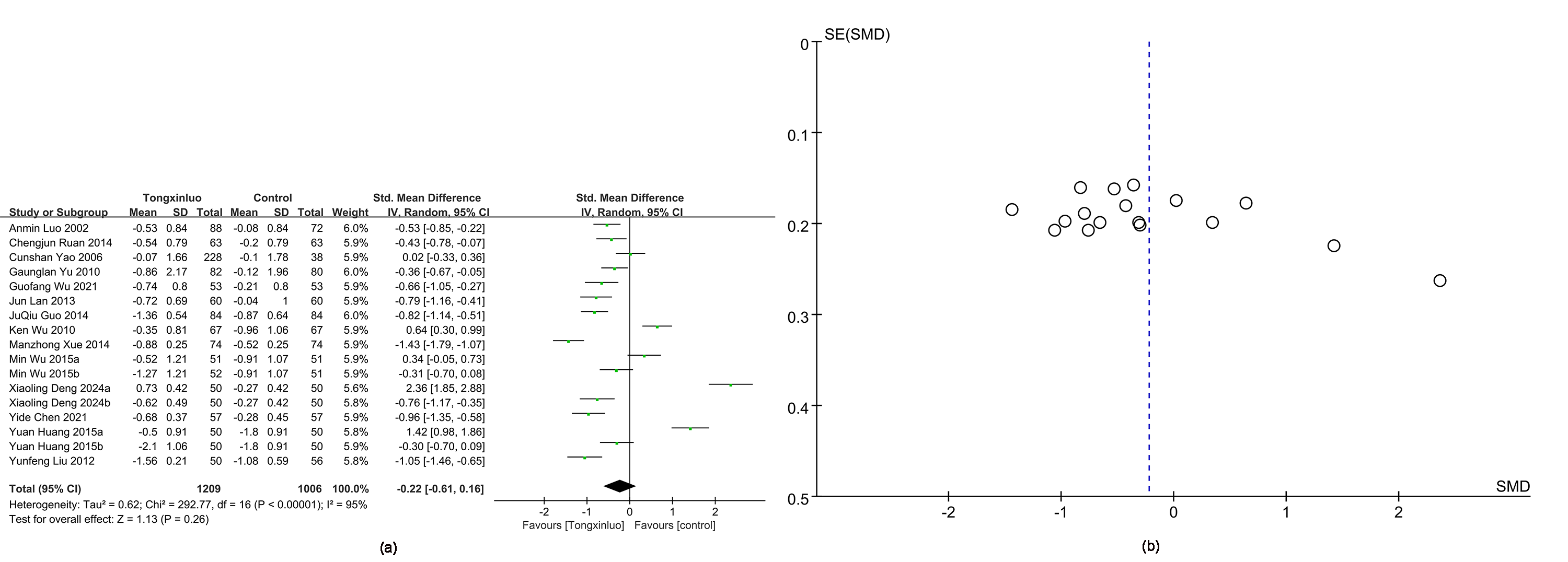 |
| --- |
| **Figure S1** Forest and funnel map of TG. (a). Forest map; (b). Funnel map. |

| 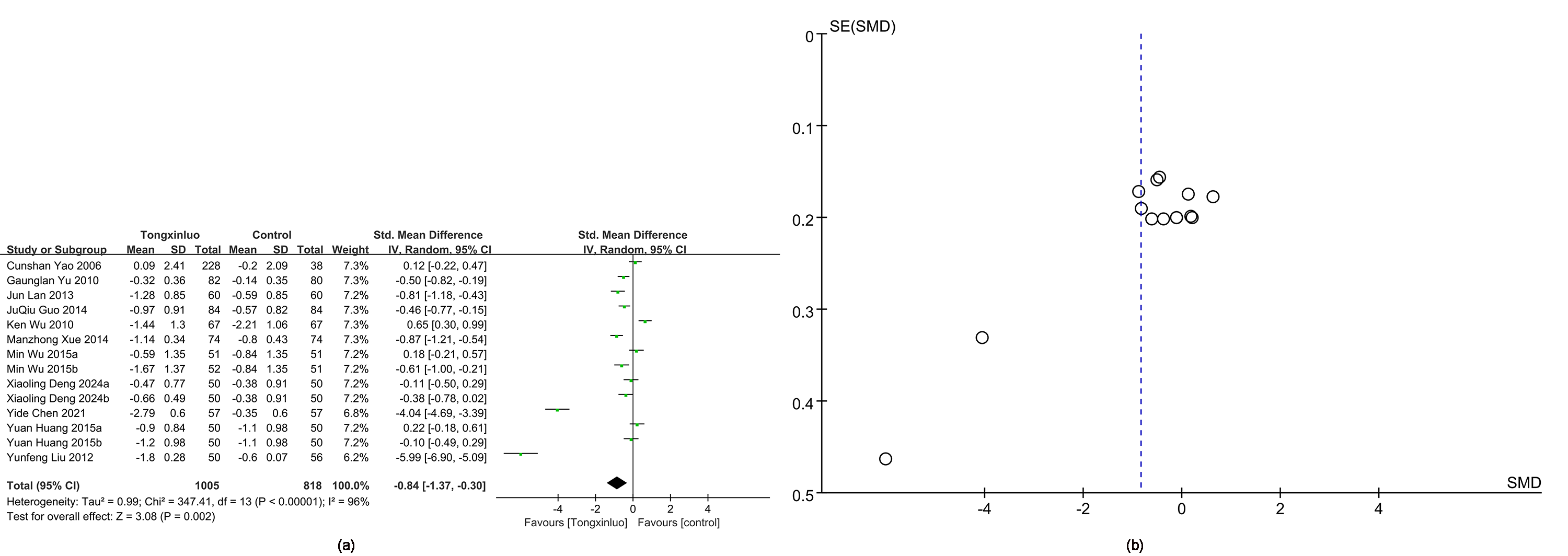 |
| --- |
| **Figure S2** Forest and funnel map of TDL. (a). Forest map; (b). Funnel map. |

| 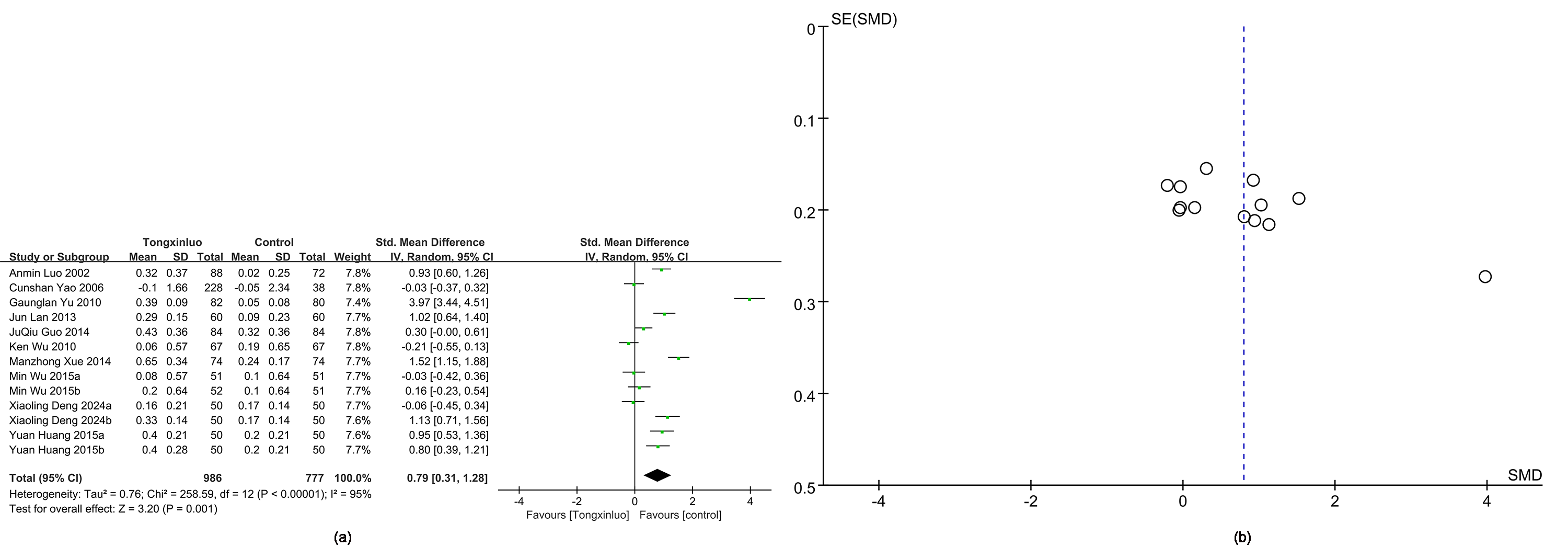 |
| --- |
| **Figure S3** Forest and funnel map of HDL. (a). Forest map; (b). Funnel map. |

| 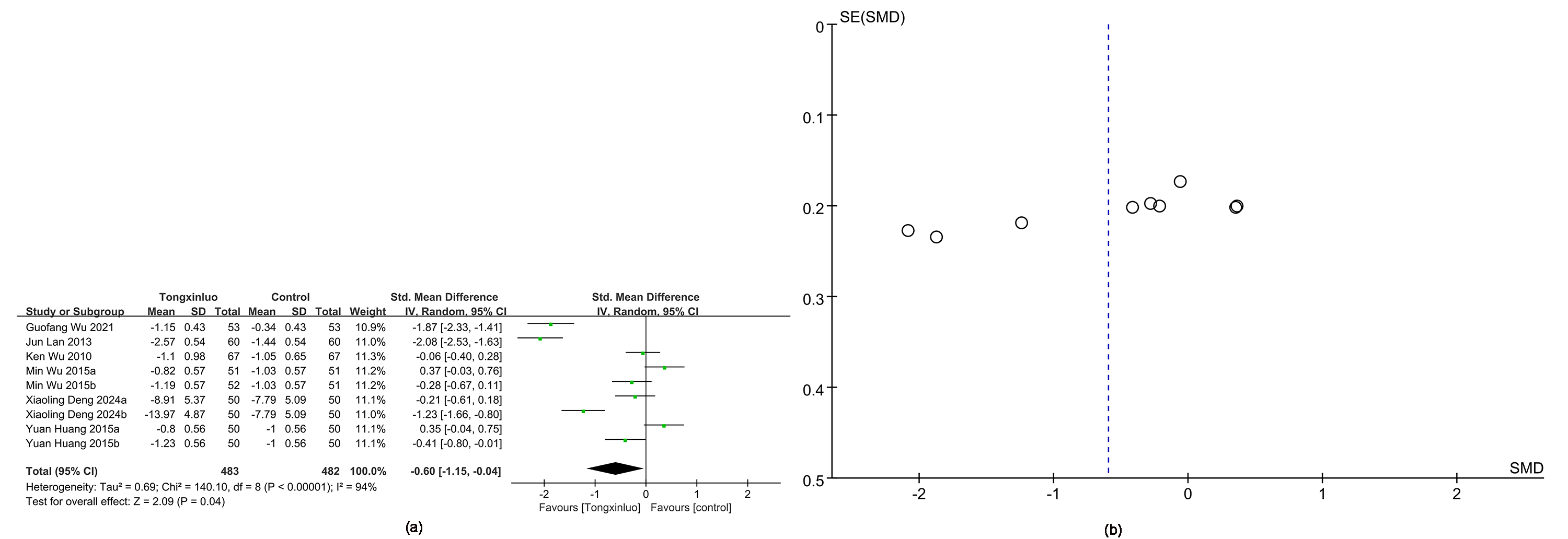 |
| --- |
| **Figure S4** Forest and funnel map of **TNF-α**. (a). Forest map; (b). Funnel map. |

| 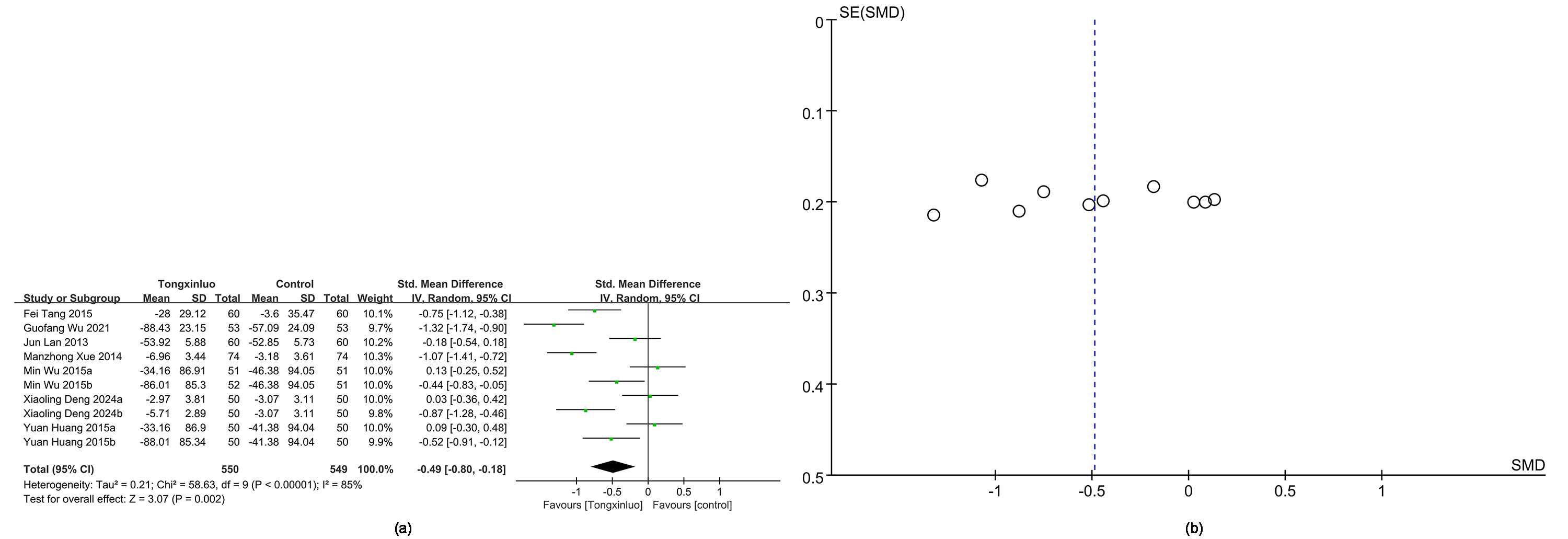 |
| --- |
| **Figure S5** Forest and funnel map of **IL-6**. (a). Forest map; (b). Funnel map. |

| 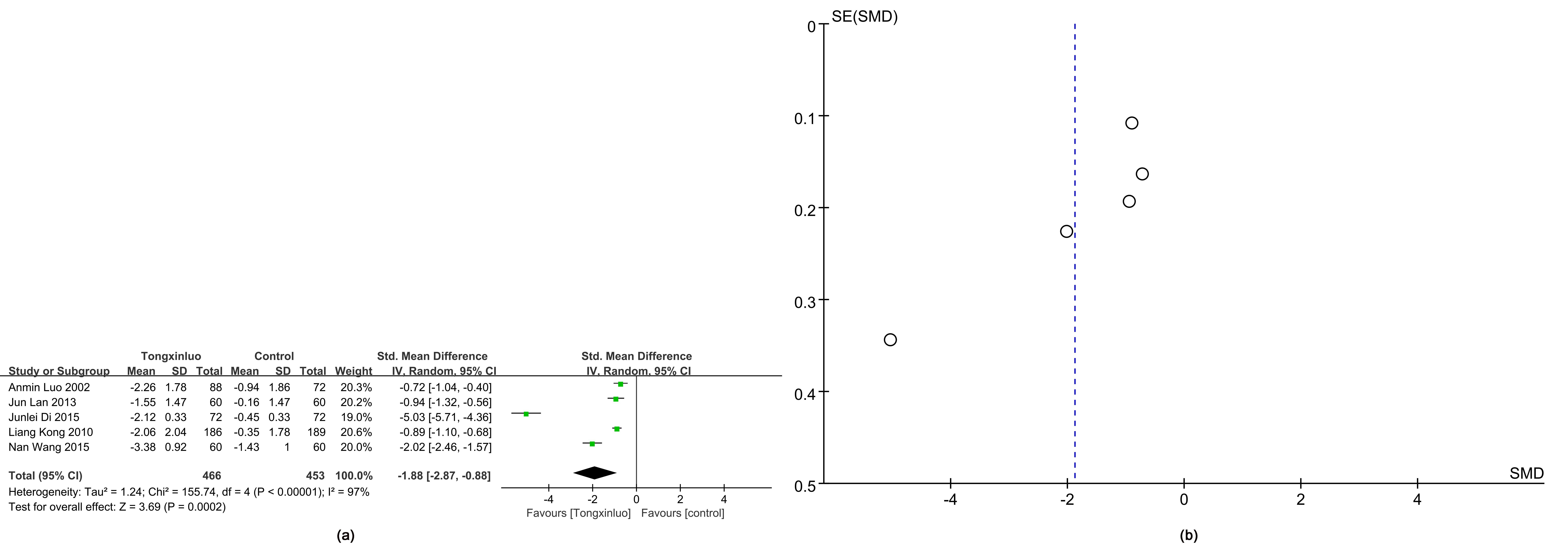 |
| --- |
| **Figure S6** Forest and funnel map of **High-Shear Whole Blood Viscosity**. (a). Forest map; (b). Funnel map. |

| 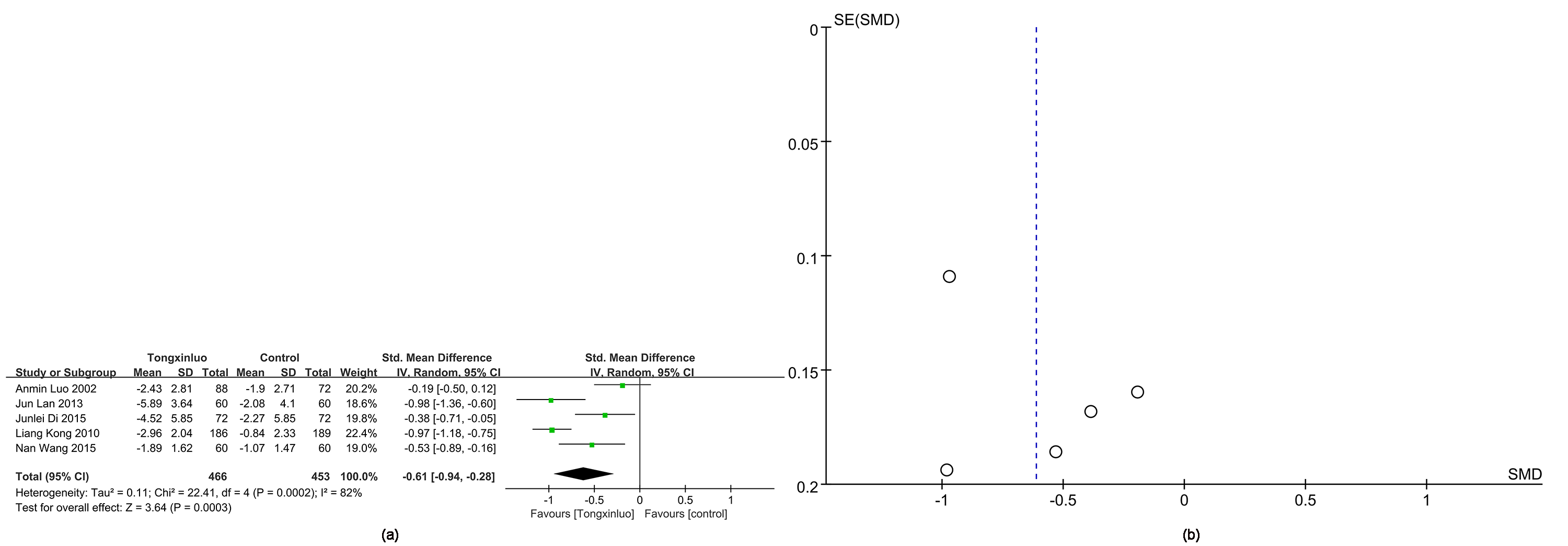 |
| --- |
| **Figure S7** Forest and funnel map of **Low-Shear Whole Blood Viscosity**. (a). Forest map; (b). Funnel map. |

| 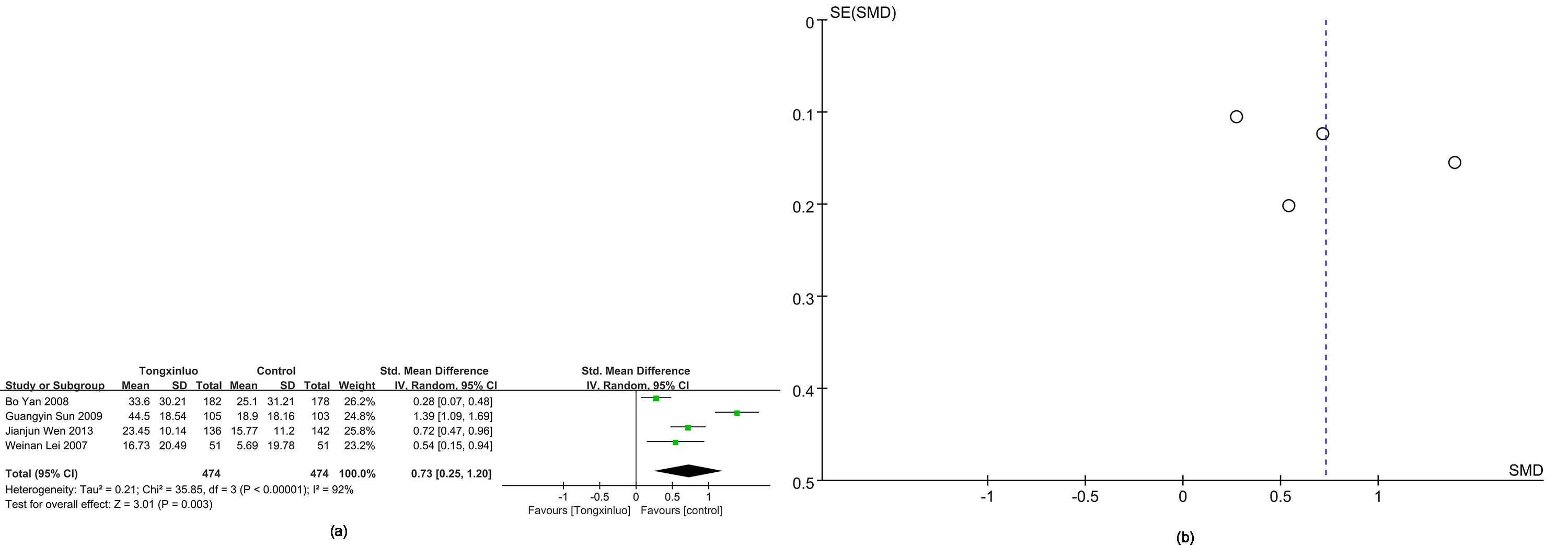 |
| --- |
| **Figure S8** Forest and funnel map of **Barthel Index Score**. (a). Forest map; (b). Funnel map. |

| 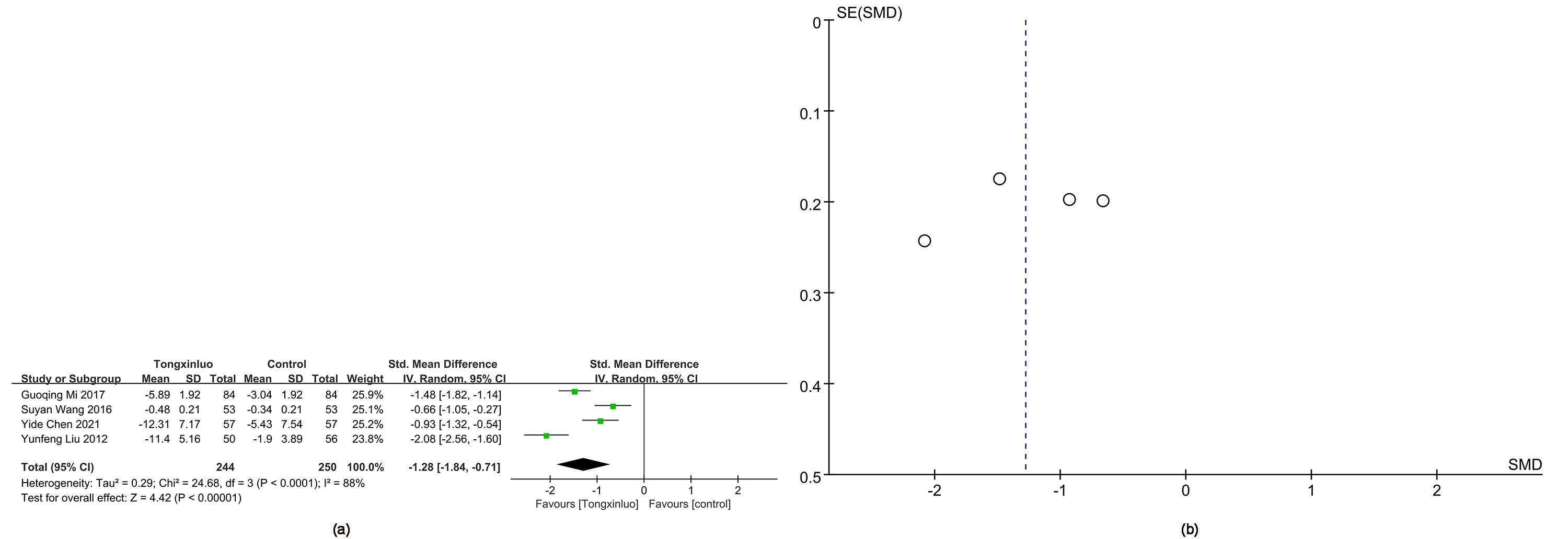 |
| --- |
| **Figure S9** Forest and funnel map of **Plaque Area**. (a). Forest map; (b). Funnel map. |

| 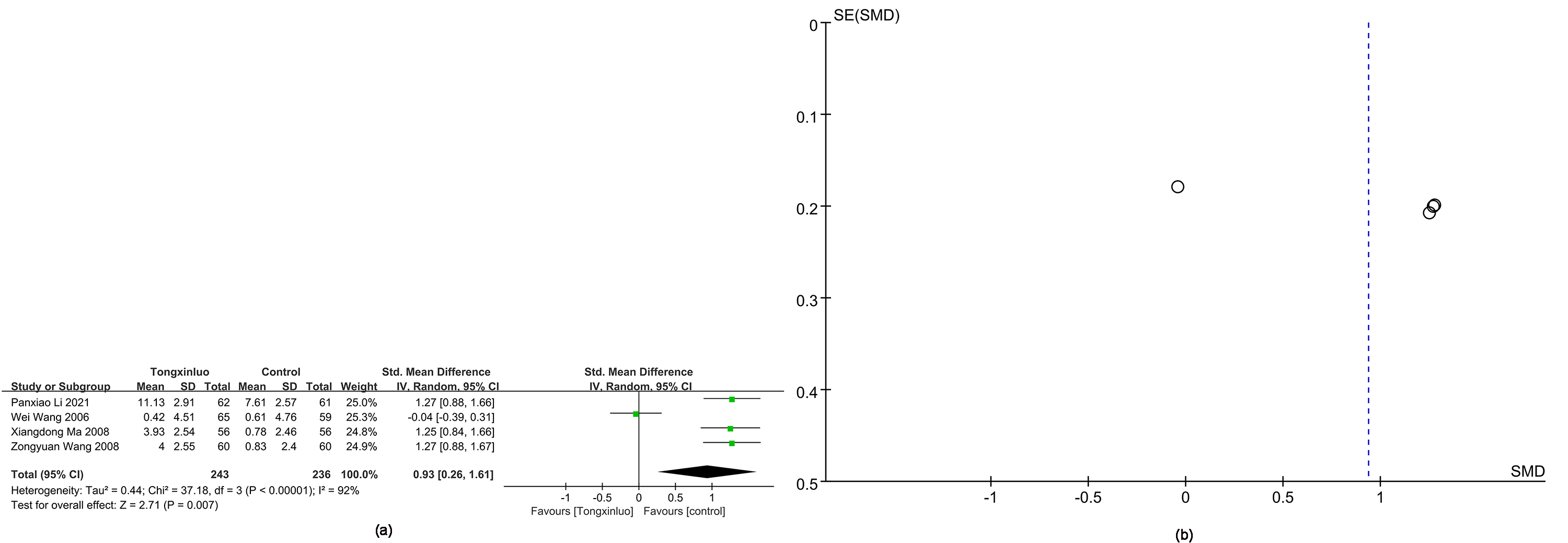 |
| --- |
| **Figure S10** Forest and funnel map of **MMSE Score**. (a). Forest map; (b). Funnel map. |

| 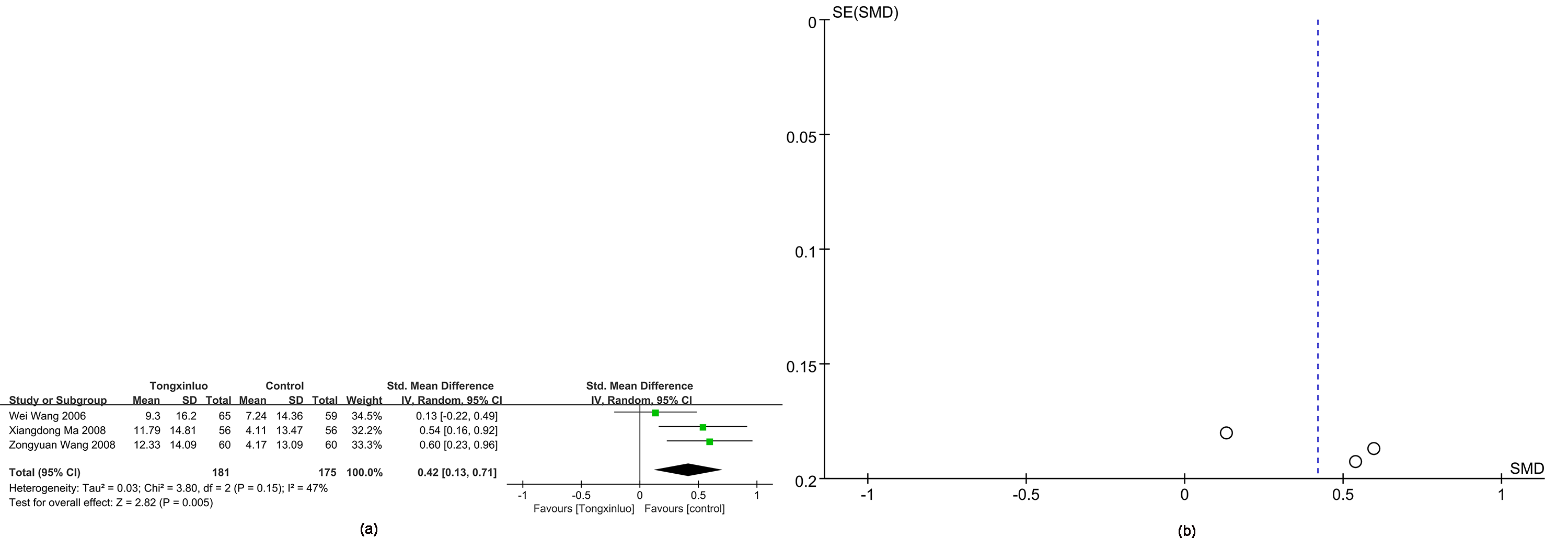 |
| --- |
| **Figure S11** Forest and funnel map of FM Score. (a). Forest map; (b). Funnel map. |

| 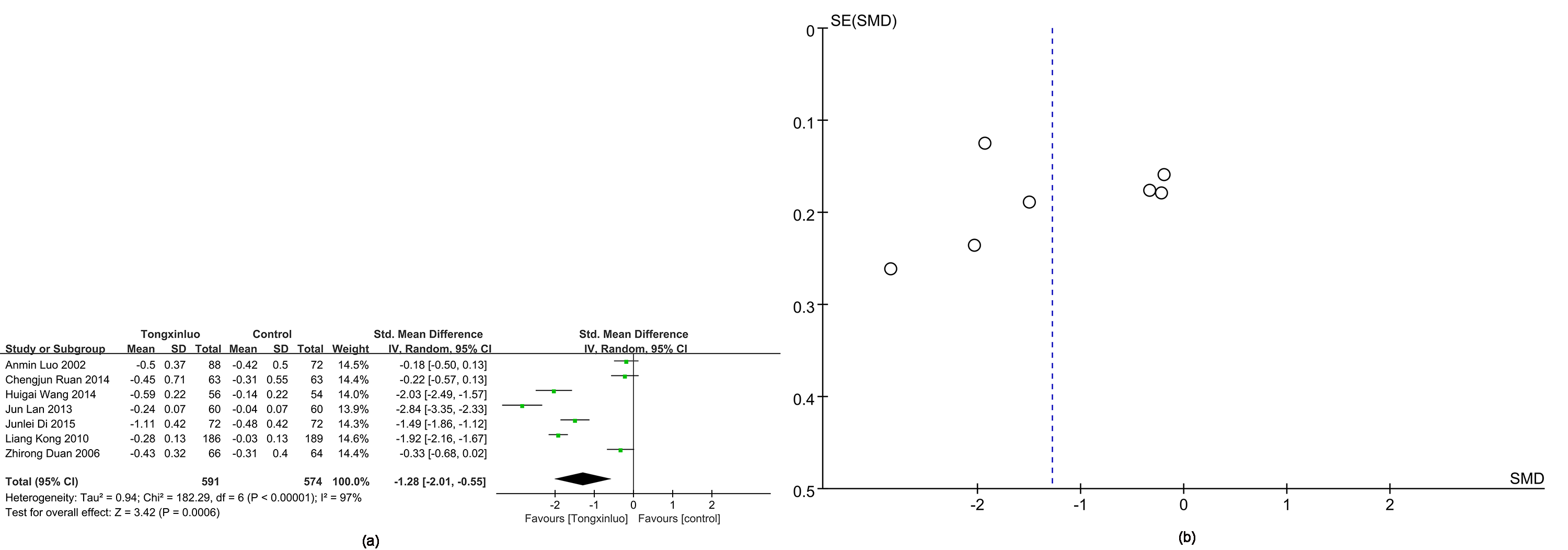 |
| --- |
| **Figure S12** Forest and funnel map of **Plasma Viscosity**. (a). Forest map; (b). Funnel map. |

| 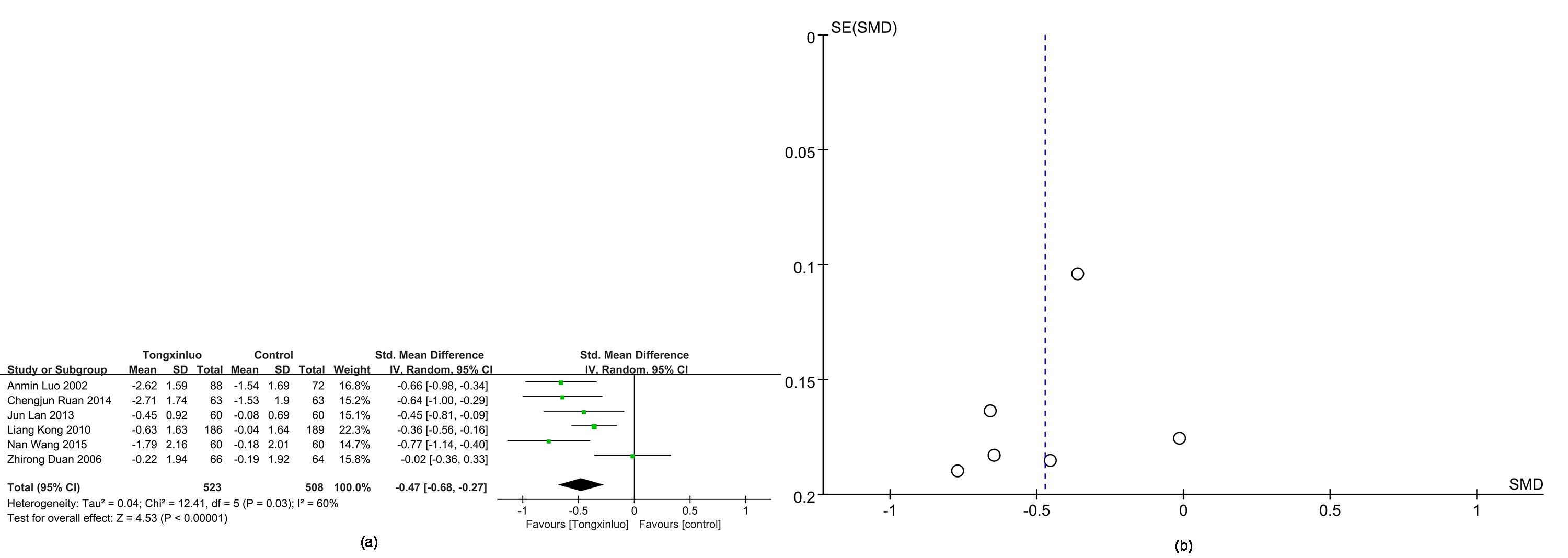 |
| --- |
| **Figure S13** Forest and funnel map of **Plasma Fibrinogen**. (a). Forest map; (b). Funnel map. |

| 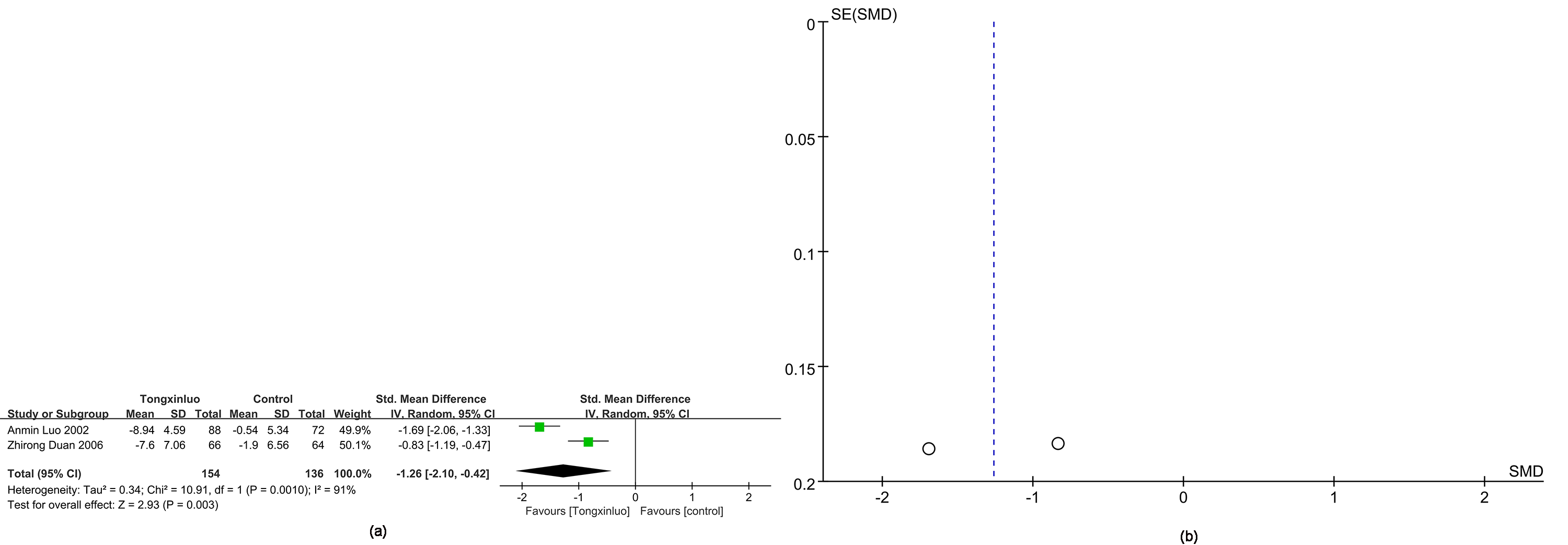 |
| --- |
| **Figure S14** Forest and funnel map of Hematocrit. (a). Forest map; (b). Funnel map. |

| 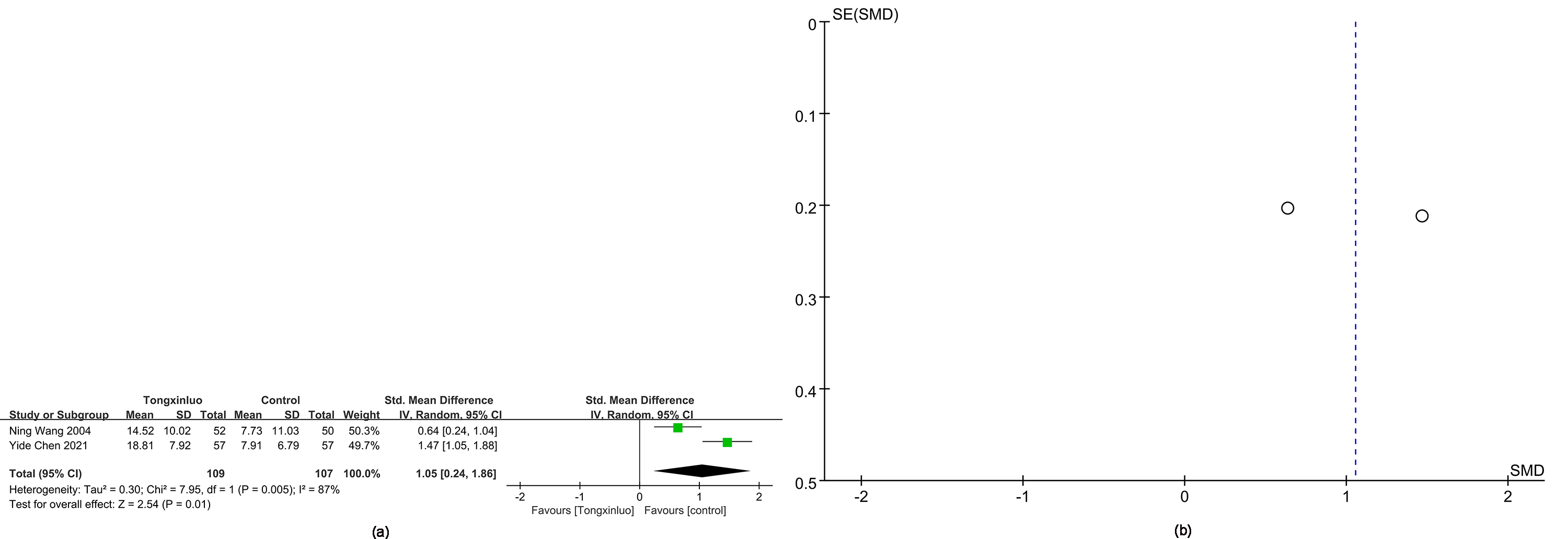 |
| --- |
| **Figure S15** Forest and funnel map of **NO**. (a). Forest map; (b). Funnel map. |

| 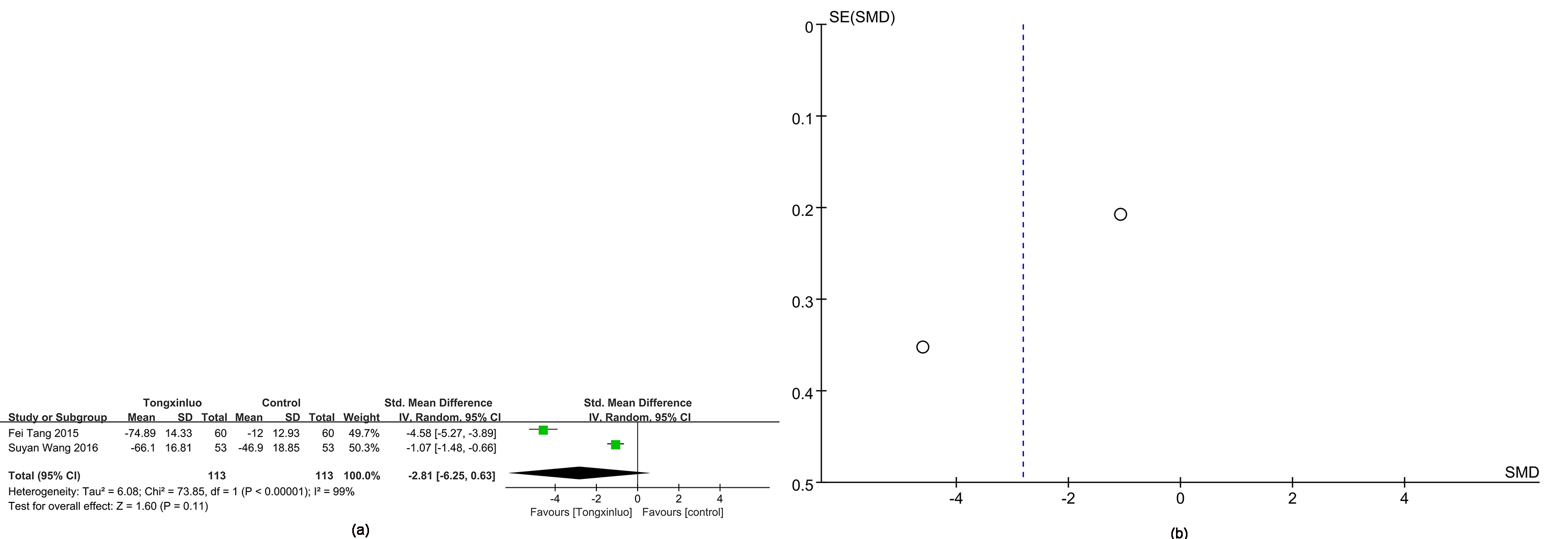 |
| --- |
| **Figure S16** Forest and funnel map of IL**-18**. (a). Forest map; (b). Funnel map. |

| 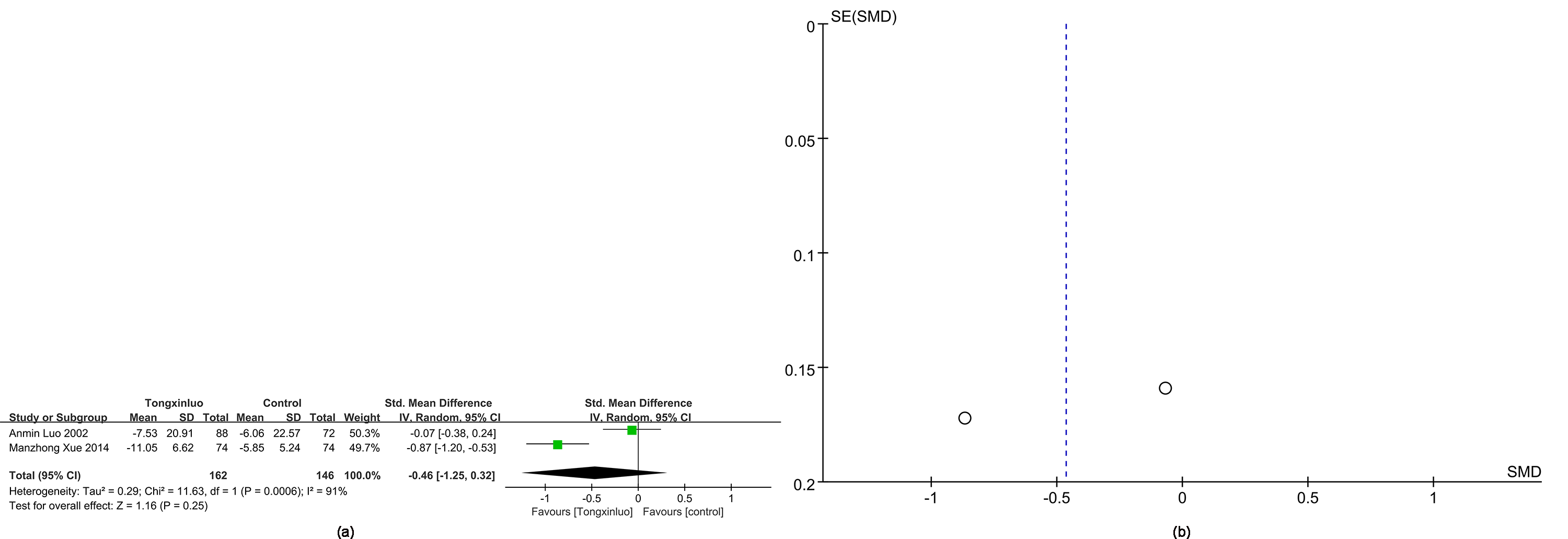 |
| --- |
| **Figure S17** Forest and funnel map of **Platelet Aggregation Function**. (a). Forest map; (b). Funnel map. |

| 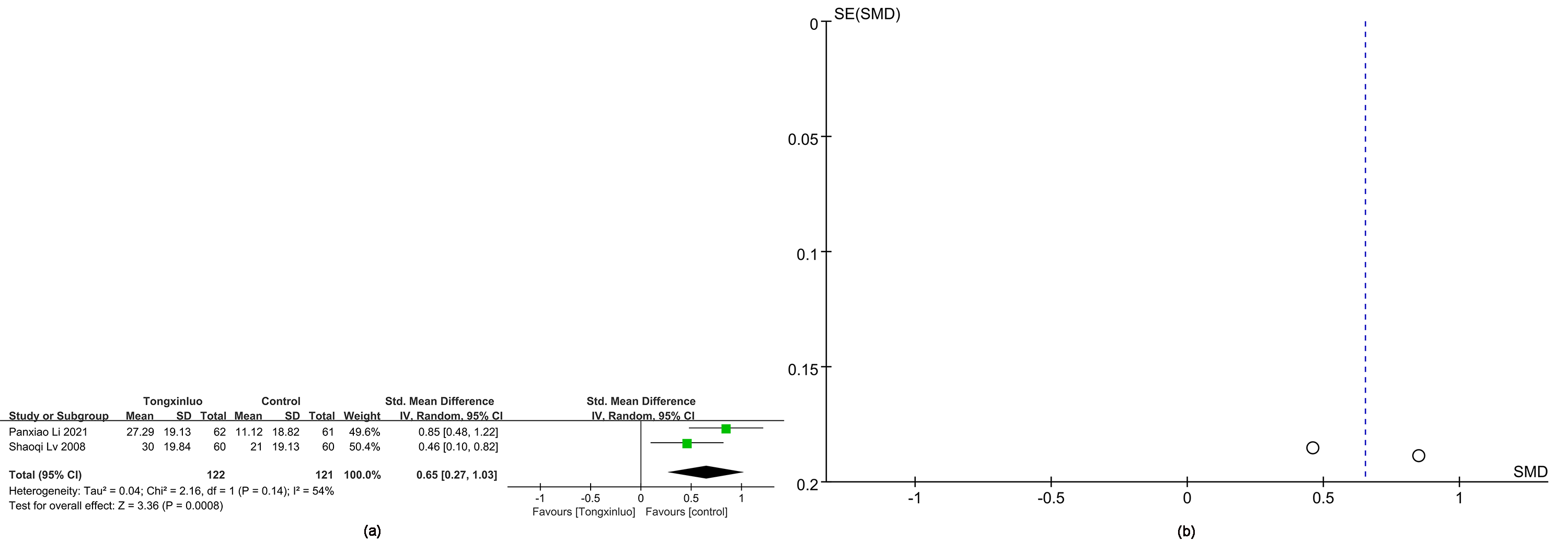 |
| --- |
| **Figure S18** Forest and funnel map of Quality of Life Scores. (a). Forest map; (b). Funnel map. |

| 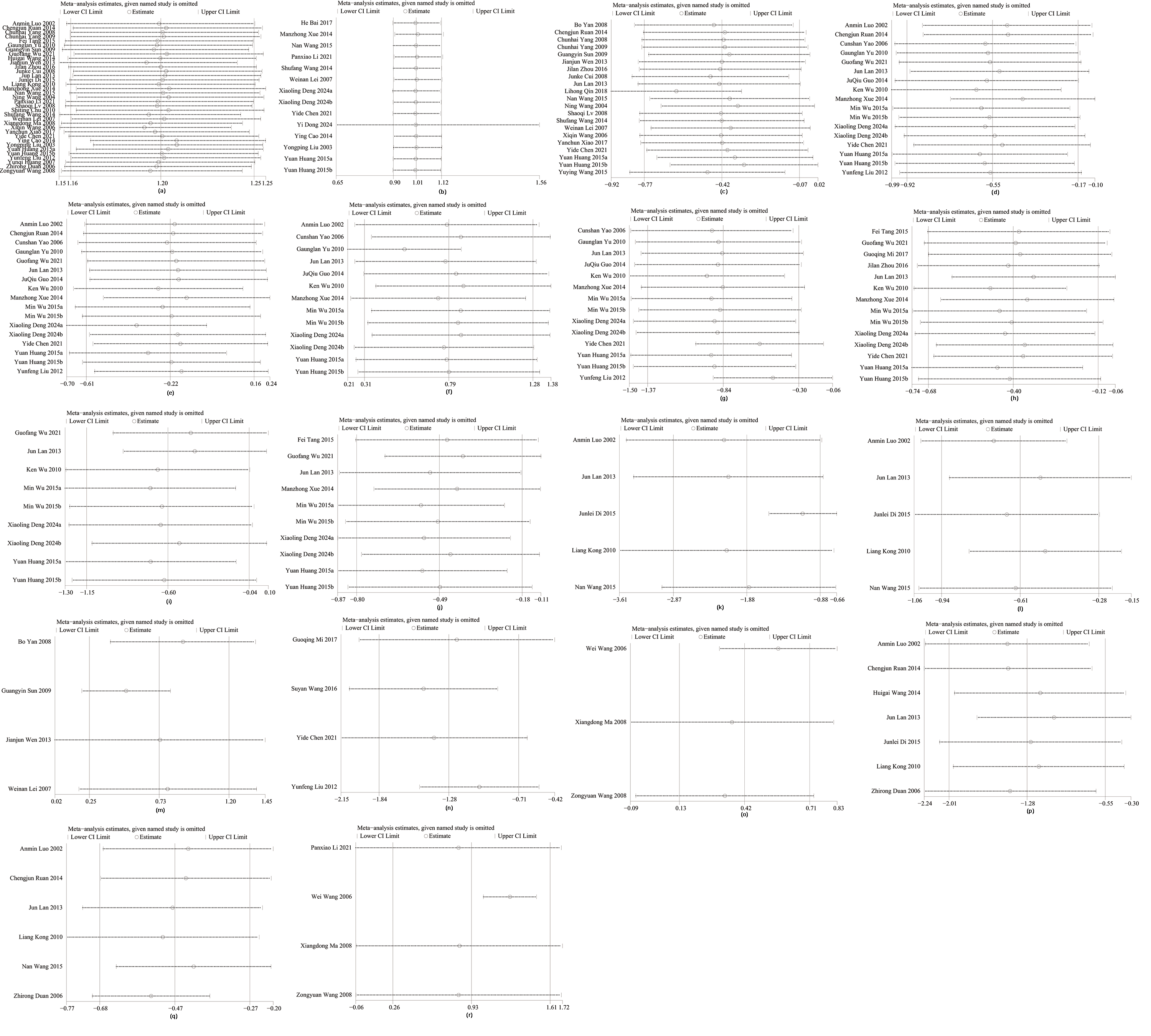 |
| --- |
| **Figure S19** Sensitivity analysis. (a). Total efficacy; (b). Adverse events; (c). NIHSS scores; (d). TC ; (e). TG; (f). HDL; (g). LDL; (h). hs-CRP; (i). TNF-α; (j). IL-6; (k). high-shear whole blood viscosity ; (l). low-shear whole blood viscosity; (m). Barthel scores; (n). plaque; (o). FM scores; (p). plasma viscosity; (q). plasma fibrinogen; (r). MMSE scores. |
